# Choice of surrogate tissue influences neonatal EWAS findings

**DOI:** 10.1186/s12916-017-0970-x

**Published:** 2017-12-05

**Authors:** Xinyi Lin, Ai Ling Teh, Li Chen, Ives Yubin Lim, Pei Fang Tan, Julia L. MacIsaac, Alexander M. Morin, Fabian Yap, Kok Hian Tan, Seang Mei Saw, Yung Seng Lee, Joanna D. Holbrook, Keith M. Godfrey, Michael J. Meaney, Michael S. Kobor, Yap Seng Chong, Peter D. Gluckman, Neerja Karnani

**Affiliations:** 1 Singapore Institute for Clinical Sciences, A*STAR, Singapore, 117609 Singapore; 2 Duke NUS Medical School, Singapore, 169857 Singapore; 3 Centre for Molecular Medicine and Therapeutics, Child and Family Research Institute, Department of Medical Genetics, University of British Columbia, Vancouver, BC V5Z 4H4 Canada; 4 KK Women’s and Children’s Hospital, Singapore, 229899 Singapore; 5 Saw Swee Hock School of Public Health, National University of Singapore, Singapore, 117597 Singapore; 6 Singapore Eye Research Institute, Singapore, 169856 Singapore; 7 Department of Paediatrics, Yong Loo Lin School of Medicine, National University of Singapore, Singapore, 119228 Singapore; 8 Division of Paediatric Endocrinology and Diabetes, Khoo Teck Puat-National University Children’s Medical Institute, National University Health System, Singapore, 119228 Singapore; 9 NIHR Biomedical Research Centre, University of Southampton, Southampton, SO16 6YD UK; 10 MRC Lifecourse Epidemiology Unit and NIHR Southampton Biomedical Research Centre, University of Southampton and University Hospital Southampton NHS Foundation Trust, Southampton, SO16 6YD UK; 11 Ludmer Centre for Neuroinformatics and Mental Health, Douglas University Mental Health Institute, McGill University, Montreal, Quebec H4H 1R3 Canada; 12 Department of Obstetrics and Gynaecology, Yong Loo Lin School of Medicine, National University of Singapore, Singapore, 119228 Singapore; 13 Centre for Human Evolution, Adaptation and Disease, Liggins Institute, University of Auckland, Auckland, 1142 New Zealand; 14 Department of Biochemistry, Yong Loo Lin School of Medicine, National University of Singapore, Singapore, 119228 Singapore

**Keywords:** Epigenome-wide association study, Tissue-specificity, DNA methylation, Prenatal factors, Genotype, Neonate

## Abstract

**Background:**

Epigenomes are tissue specific and thus the choice of surrogate tissue can play a critical role in interpreting neonatal epigenome-wide association studies (EWAS) and in their extrapolation to target tissue. To develop a better understanding of the link between tissue specificity and neonatal EWAS, and the contributions of genotype and prenatal factors, we compared genome-wide DNA methylation of cord tissue and cord blood, two of the most accessible surrogate tissues at birth.

**Methods:**

In 295 neonates, DNA methylation was profiled using Infinium HumanMethylation450 beadchip arrays. Sites of inter-individual variability in DNA methylation were mapped and compared across the two surrogate tissues at birth, i.e., cord tissue and cord blood. To ascertain the similarity to target tissues, DNA methylation profiles of surrogate tissues were compared to 25 primary tissues/cell types mapped under the Epigenome Roadmap project. Tissue-specific influences of genotype on the variable CpGs were also analyzed. Finally, to interrogate the impact of the in utero environment, EWAS on 45 prenatal factors were performed and compared across the surrogate tissues.

**Results:**

Neonatal EWAS results were tissue specific. In comparison to cord blood, cord tissue showed higher inter-individual variability in the epigenome, with a lower proportion of CpGs influenced by genotype. Both neonatal tissues were good surrogates for target tissues of mesodermal origin. They also showed distinct phenotypic associations, with effect sizes of the overlapping CpGs being in the same order of magnitude.

**Conclusions:**

The inter-relationship between genetics, prenatal factors and epigenetics is tissue specific, and requires careful consideration in designing and interpreting future neonatal EWAS.

**Trial registration:**

This birth cohort is a prospective observational study, designed to study the developmental origins of health and disease, and was retrospectively registered on 1 July 2010 under the identifier NCT01174875.

**Electronic supplementary material:**

The online version of this article (doi:10.1186/s12916-017-0970-x) contains supplementary material, which is available to authorized users.

## Background

Epigenetic processes, such as DNA methylation, are important regulators of gene expression and thus play a vital role in human development and disease. Inter-individual variation in infant DNA methylomes can arise from genetic [1, 2], environmental [3], or stochastic perturbations [4]. Epigenome-wide association studies (EWAS) using neonate tissues can help interrogate the inter-relationship between these factors and enhance our understanding of the biological mechanisms underpinning disease predisposition and progression. Further, they are also instrumental in identifying diagnostic and prognostic biomarkers.

Epigenomes are tissue specific [5] and thus the choice of neonatal tissue is an important consideration in designing a neonatal EWAS. However, the target tissues of direct relevance to the outcome of interest are often impossible or extremely difficult to collect. As an alternate approach, surrogate tissues, such as cord blood, cord tissue, placenta, or buccal epithelium, are used as proxies for target tissues. A number of studies have compared DNA methylation markers across different surrogate neonatal tissues. For example, in a twin-study, Gordon et al. [4] reported the influence of genetic factors on a subset of variable CpGs to be higher in cord blood than placenta or human umbilical vein endothelial cells (HUVEC). Armstrong et al. [6] compared the DNA methylation status of seven candidate gene loci and repeat sequences (*LINE*-1 and ALUYb7) in the genome across three infant tissues (cord blood, placenta, and early infancy buccal epithelium), and reported tissue-specific differences in DNA methylation levels for most of the tested loci. Previous studies have also observed less concordance in the DNA methylation–prenatal factor associations performed on different infant tissues. For example, Lesseur et al. [7] reported significant associations between leptin DNA methylation and genetic variation, weight-for-gestational-age, maternal adiposity, and maternal smoking in cord blood, but not in placenta. Similarly, Novakovic et al. [8] found associations between intrauterine cigarette smoke exposure and aryl hydrocarbon receptor repressor DNA methylation in both cord blood and infant blood at 18 months, but not in placenta and buccal epithelium at birth. Likewise, Nomura et al. [9] reported links between maternal gestational diabetes, preeclampsia, and obesity with global DNA methylation levels in placenta but not in cord blood. Contrary to these tissue-specific DNA methylation–prenatal factor findings, Ruchat et al. [10] found a greater than 25% overlap in the genes differentially methylated in response to maternal gestational diabetes in placenta and cord blood.

Even though previous comparisons have advanced our understanding of the relationship of neonate DNA methylomes with genetic variation, prenatal exposure perturbations, and tissue specificity, there are still outstanding questions that remain unanswered in context of a neonatal EWAS. First, previous DNA methylation–prenatal factor investigations thus far have been limited in statistical power and coverage of the epigenome as they have been conducted on small sample sizes (N < 100) and/or have investigated only a few candidate genes or repeat regions. It remains unclear how genome-wide site-specific DNA methylation from different neonate tissues would compare in larger sample sizes. Second, previous comparisons have mostly scrutinized DNA methylation profiles from cord blood, buccal epithelium, and placenta, while there have been fewer reports from cord tissue. Additionally, there is limited data on the utility of cord tissue versus cord blood as a surrogate tissue in a neonate EWAS. Third, previous reports have largely restricted their comparisons to either a few prenatal factors or just genetic variation. These single-faceted investigations provide a useful yet incomplete picture of the complex associations between genetics, prenatal factors, and epigenetics across different neonatal tissues.

To address the limitations of previous studies, we provide a large-sample epigenome-wide comparison of genome-wide DNA methylation from two neonatal tissues, and their association with genetic and prenatal factor influences. To accomplish this, we first measured and compared the inter-individual variation in DNA methylation of the two neonatal tissues. Second, by comparing the DNA methylation profiles of these surrogate tissues with the DNA methylation profiles of different fetal and adult tissues mapped under the Epigenome Roadmap project, we determined the target tissues that these surrogate tissues can proxy for. Third, we investigated the extent to which inter-individual variation in these tissues can be explained by genetic factors. Finally, we also examined the extent to which inter-individual variation in these surrogate tissues can be explained by prenatal factors by comparing the neonate EWAS results from prenatal factors.

## Methods

### Study population

Mother–offspring dyads were prospectively recruited as part of the Growing Up in Singapore Towards Healthy Outcomes (GUSTO) birth cohort study, which has been previously described [11]. Pregnant women in their first trimester of pregnancy and of at least 18 years of age were recruited from the two major public hospitals with obstetric services in Singapore, namely the KK Women’s and Children’s Hospital (KKH) and the National University Hospital (NUH). To be eligible, participants had to hold Singapore citizenship or permanent residency, or intent to reside in Singapore for the next 5 years, were of Chinese, Malay or Indian ethnicity, had homogeneous parental ethnic background, and had the intention to deliver at either NUH or KKH. Women with significant health conditions such as those who were on chemotherapy or psychotropic drugs were excluded from the study. The present analysis was restricted to live singleton full-term births, with an Apgar score of at least nine, and with infant genotype and DNA methylation data (cord tissue and cord blood) (Additional file 1: Figure A1). Gestational age (GA) was determined by ultrasonography in the first trimester. Child sex was extracted from the medical records.

### Prenatal factors – demographics, maternal smoking, and alcohol use

At enrolment, interviewer-administered questionnaires were used to collect information on maternal age and education. An interviewer-administered questionnaire was also conducted at 26–28 weeks’ gestation to obtain information on maternal occupational activity during pregnancy, maternal alcohol usage before and during pregnancy, and maternal smoking behavior before and during pregnancy. Parity (birth order) was extracted from medical records.

### Prenatal factors – maternal mood

The Spielberger State-Trait Anxiety Inventory (STAI) scale and the Edinburgh Postnatal Depression Scale (EPDS) were used to assess maternal anxiety and depression, respectively, at 26–28 weeks’ gestation. The STAI instrument contains 40 items scored on a 4-point Likert scale, with 20 items ascertaining the trait measure and 20 items ascertaining the state measure. The trait measure is a reflection of a more stable personality characteristic, such as an anxious personality, while the state measure is a reflection of transient characteristics of anxiety such as anxiety disorders. The EPDS instrument assesses 21 common depressive symptoms experienced over the past week.

### Prenatal factors – maternal metabolic/anthropometry

Pre-pregnancy weight was self-reported during study recruitment in the first trimester of pregnancy. Maternal height and weight were measured at 26–28 weeks’ gestation. Gestational weight gain (GWG) was calculated as the difference between pre-pregnancy and 26–28 week weights. Maternal pre-pregnancy BMI (ppBMI) was derived as pre-pregnancy weight divided by height squared. Maternal glucose levels (2-h post-glucose (2-h PG) and fasting plasma glucose (FPG)) were ascertained at 26–28 weeks using an oral glucose tolerance test of 75 g after an overnight fast (8–14 h). Maternal peripheral systolic blood pressure (SBP) and diastolic blood pressure (DBP) at 26–28 weeks’ gestation were measured from the brachial artery at 30- to 60-second intervals.

### Prenatal factors – maternal fatty acids and vitamins

Maternal plasma fatty acids were measured using serum drawn at 26–28 weeks’ gestation. The fatty acids were expressed as percentage contribution to total plasma phosphatidylcholine fatty acid. We investigated the total n-6 polyunsaturated fatty acids (PUFAs), the total n-3 PUFAs, the total PUFAs (n-6 PUFAs + n-3 PUFAs), the total monounsaturated fatty acids (MUFAs), and the total saturated fatty acids (SFAs). We also investigated individual saturated fatty acids myristic acid, palmitic acid, and stearic acid; monounsaturated fatty acids oleic acid and gondoic acid; n-3 PUFAs eicosatetraenoic acid (ETA), eicosapentaenoic acid (EPA), docosapentaenoic acid (DPA), and docosahexaenoic acid (DHA); and n-6 PUFAs linoleic acid, dihomo-gamma-linolenic acid (DGLA), and n-6 arachidonic acid (AA). Finally, the n-6:n-3 PUFA ratio, namely AA:DHA ratio, AA:EPA ratio, DHA:DPA ratio, and AA:(DHA + EPA) ratio were also assessed. Maternal micronutrient levels, including vitamin D, vitamin B6, vitamin B12, and folate, were tested using serum drawn at 26–28 weeks’ gestation.

### Tissue collection and processing

#### Cord blood

Up to 40 mL of cord blood was collected from infant umbilical cords within 4 h post-delivery, either by directly dripping into EDTA tubes for normal deliveries, or extracted through a syringe for cords delivered through cesarean section deliveries, then stored in EDTA tubes. Blood samples were then centrifuged at 3000 *g* at 4 °C for 5 min to separate the blood into three distinct layers – plasma, buffy coat, and erythrocytes. The top plasma layer was then carefully extracted (without disturbing the buffy coat), followed by extraction of the buffy coat layer. The buffy coat was stored at −80 °C. DNA extraction from the buffy coat was performed using QIAsymphony DNA kits as per the manufacturer’s instructions.

#### Cord tissue

After the extraction of cord blood, sections of umbilical cord tissue (~2 cm per section) were collected and cleaned with phosphate buffer saline solution. Each section was then cut into smaller pieces with a clean scalpel and stored into 2 mL cryovials. The cord samples were then snap frozen in liquid nitrogen and stored at −80 °C until subsequent DNA extraction. For DNA extraction, frozen umbilical cords were pulverized with a mortar and pestle, weighed, and allowed to equilibrate to room temperature before treatment with 10 U/mL hydraluronidase enzyme, ensuring that all tissue was submerged in the enzyme solution. Cord samples were then incubated at 37 °C for 30 min on a shaker (150 rpm) in an incubator. Then, 250 μL of Tris-NaCl-EDTA-SDS solution was added before the tissue was homogenized six times (10 seconds each cycle) using a Xiril Dispomix homogenizer. Samples were then pulse spun to pellet the tissue prior to adding proteinase K, and incubated overnight at 55 °C. NaCl (250 μL, 5 M) was added and the contents of the tube were mixed. Samples were centrifuged at 3500 *g* for 20 min and the supernatant transferred to a fresh tube. An equal volume of 100% ethanol was added to the supernatant with gentle mixing to allow DNA to precipitate. DNA was spooled and transferred to a fresh tube containing 500 μL of water and 5 μL of RNase A solution. Samples were then incubated at 55 °C for 30 min to remove the RNA. The DNA solution was transferred to MaXtract tubes, where an equal volume of phenol/chloroform was added with gentle mixing, and then centrifuged at 20,000 *g* for 10 min. The top aqueous layer was extracted and the phenol/chloroform wash step repeated. The final top aqueous layer was extracted and a 10% volume of 3 M NaAc (pH 5.2) and a 200% volume of 100% ethanol was added, gently mixed, and allowed to precipitate DNA for 10 min at −80 °C. This solution was centrifuged at 20,000 *g* for 10 min to pellet down the DNA. The supernatant was removed and the DNA pellet was washed with 70% ethanol, spun down again, and the supernatant removed. The DNA pellet was air dried to the point of translucency and re-suspended in 100 μL TE buffer to dissolve the DNA.

### DNA methylation data – infant cord tissue, infant cord blood

Profiling and downstream processing of DNA methylation data from both tissues (infant umbilical cord tissue, infant cord blood) were conducted separately but followed similar procedures. We used the Infinium HumanMethylation450 array, following standard protocol and processed the data using an in-house quality control procedure [12]. Raw DNA methylation beta values were exported from GenomeStudio^TM^. Probes with less than three beads for either the methylated or unmethylated channel or with a detection *P* value above 0.01 were set to missing. Probes on sex chromosomes were removed. We further retained probes that had non-missingness in all samples. Color adjustment and normalization of Type 1 and 2 probes was performed. To assess the presence and impact of technical variables, we performed a principal component analysis (PCA) on the raw DNA methylation data and regressed the principal components against technical variables, including (1) chip-set (8 chips containing 96 samples per chip-set for cord tissue; 15 chips containing 180 samples per chip-set for cord blood), (2) chip (12 samples per chip), (3) chip position, (4) bisulfite conversion batch (96 samples per plate), and (5) DNA extraction batch (cord tissue only). Samples within a chip (12 samples) were nested within a chip-set (96 or 180 samples), but samples in the same bisulfite conversion plate (96 samples) were not necessarily nested within a chip-set. The top principal components from the PCA of raw DNA methylation data were most strongly associated with the chip variable. We thus used COMBAT [13] to adjust for chip effects. DNA methylation beta values were first converted to M-values before applying COMBAT to remove chip effects and the COMBAT-corrected DNA methylation values were transformed back to beta-values. We then conducted another PCA on the COMBAT-corrected dataset. Position on chip, bisulfite conversion batch, chip-set (for cord blood only), and DNA extraction batch (for cord tissue only) were associated with the top principal components and these were adjusted for as covariates in all regression models. Because we did not have complete information for some of the potential sources, to allow for the possibility of other technical artifacts besides the ones considered here, we used surrogate variable analysis [14, 15] to estimate sources of batch effects directly from the DNA methylation data (surrogate variables). The surrogate variables can also help account for cell type composition. We conducted additional sensitivity analyses, where we repeated all analyses adjusting for surrogate variables from the surrogate variable analysis. Finally, cross-hybridizing probes [16, 17], CpGs located at single nucleotide polymorphisms (SNPs), and CpGs with multi-modal distribution were excluded from the analysis. After quality control filtering, 239,560 CpGs that passed quality control in both datasets were available for analysis. For infant cord tissue, cellular proportions for fibroblasts, B-cells, and T-cells were estimated [18] using a reference panel (accession number EGAD00010000460) [19], and their principal components were adjusted as covariates in the regression models. Likewise, for infant cord blood, we used the reference panel reported by Bakulski et al. [20] to obtain estimated cellular proportions in nucleated red blood cells, granulocytes, monocytes, natural killer cells, B-cells, CD4+ T-cells, and CD8+ T-cells. Their principal components were then adjusted as covariates in all regression models. As we have previously observed that the association of cellular proportions with prenatal factors/DNA methylation could be ethnicity dependent, interaction terms between (principal components of) cellular proportions and ethnicity were included as covariates in all regression models (in addition to their main effects).

### Genotype data

Genotyping for infant was performed using the Illumina OmniExpressExome array. Non-autosomal SNPs as well as SNPs with call rates less than 95% or minor allele frequency less than 10% or failed Hardy–Weinberg equilibrium were excluded. PCA was used to confirm self-reported ethnicity/ancestry. Samples with a call rate less than 99%, cryptic relatedness, or sex/ethnic discrepancies were excluded. Alleles were expressed at the positive strand of the human build (hg19). After quality control filtering, 487,176 SNPs that passed quality control were available for analysis.

### Statistical analysis

#### CpG sites that showed inter-individual variation in each infant tissue

We first quantified the number of CpGs that showed inter-individual variation in each tissue (infant umbilical cord tissue, infant cord blood). For each CpG in each tissue, a CpG was defined to show inter-individual variation if the DNA methylation range (maximum–minimum, excluding outliers) was greater than 10% and the DNA methylation 99th percentile–1st percentile was greater than 5%. The CpGs were segregated into four distinct categories as (1) CpGs which showed inter-individual variation in both tissues, (2) CpGs which showed inter-individual variation only in infant cord blood, (3) CpGs which showed inter-individual variation only in infant cord tissue, and (4) CpGs which did not show inter-individual variation in either tissue. Each group of CpGs was annotated in terms of their genomic features (promoter, 5′-UTR, exon, intron, 3′-UTR, TTS, and intergenic) and CpG content (island, shores, shelves, open seas) using *Homer annotatePeaks* function (hg19). We also annotated the genomic location of each group of CpGs in the enhancers predicted by either the Encyclopedia of DNA Elements (ENCODE) consortium [21] or the Functional Annotation of the Mammalian Genome (FANTOM) consortium [22]. For predicted enhancers from ENCODE, we used the annotation that was included in the Infinium HumanMethylation450 manifest file. The FANTOM5-predicted enhancer annotation was obtained by using FANTOM5 Phase 1 and Phase 2 data.

#### Hierarchical clustering

Two sets of hierarchical clustering analyses were performed. First, we performed hierarchical clustering using DNA methylation data of all the study (GUSTO) samples (295 infant cord tissue samples, 295 infant cord blood samples). The clustering was conducted using all CpGs that passed quality control filtering. The clustering analysis confirmed that all 295 infant cord tissue samples clustered together as did all 295 infant cord blood samples (Additional file 1: Figure B2). Second, we performed hierarchical clustering of the study (GUSTO) samples with 25 primary tissues/cells profiled using reduced representation bisulfite sequencing in the Epigenome Roadmap project [5]. For each of the GUSTO tissues (infant cord tissue, infant cord blood), the median value across all 295 samples was used to represent each CpG in each tissue. For DNA methylation data generated by the Epigenome Roadmap project, we retained only DNA methylation sites that had a minimum reads coverage of 30X and reads from both strands were combined. The hierarchical clustering was performed using CpG sites that passed quality control filtering in the GUSTO tissues (infant cord tissue, infant cord blood), were non-missing in at least 10 out of the 25 Epigenome Roadmap samples, and had interquartile range greater than 10% across different Epigenome Roadmap tissues/cells. We also computed the Spearman correlation between each GUSTO sample/tissue and each Epigenome Roadmap tissue/cell.

#### Genetic influences on DNA methylation

We determined if inter-individual variation in DNA methylation in each tissue could be explained by genotype. CpGs whose inter-individual variation in DNA methylation could be explained by SNPs were defined to be influenced by genetic factors (SNPs) or genotype-associated factors. We regressed each CpG that showed inter-individual variation in each tissue, against all *cis*-SNPs (all SNPs that resided on the same chromosome as the CpG), using an additive genotype model. To help increase the precision of the estimates of effect sizes in assessing the association between genotype and DNA methylation, we adjusted for child sex, GA, ethnicity, cellular proportions, bisulfite conversion batch, hospital, DNA extraction batch (for cord tissue only), chip-set (for cord blood only), and chip position, as these variables were associated with the top principal components from a PCA of the COMBAT-corrected DNA methylation dataset. We also included interactions between ethnicity and cellular proportions in the regression models. DNA methylation outliers were truncated to the boundary (next possible) value. For each CpG, we reported the most significant association (smallest *P* value) between the CpG and *cis*-SNPs. A CpG was defined to be genotype-associated or have its inter-individual variation explained by SNPs if the most significant association between the CpG and *cis*-SNPs attained a *P* value < 5 × 10^–8^, the Bonferroni threshold typically used in genome-wide association studies (corresponding to testing for approximately 10^6^ independent SNPs at a family-wise Type 1 error rate of 0.05). For each tissue, we report the number and percentage of genotype-associated CpGs out of all CpGs that showed inter-individual variation in the tissue. We also report whether the CpG was genotype-associated in the other tissue.

#### Prenatal factor influences on DNA methylation

We investigated whether inter-individual variation in DNA methylation in each tissue could be explained by prenatal factors. Linear regression models were used to study the association of DNA methylation with each of the 45 prenatal factor variables. To help increase the precision of the estimates of effect sizes in assessing the association between prenatal factors and DNA methylation, we adjusted for child sex, GA, ethnicity, cellular proportions, bisulfite conversion batch, hospital, DNA extraction batch (for cord tissue only), chip-set (for cord blood only), and chip position, as these variables were associated with the top principal components from a PCA of the COMBAT-corrected DNA methylation dataset. We also included interactions between ethnicity and cellular proportions in the regression models. To ensure that results were robust to the presence of outliers, outliers in DNA methylation and continuous prenatal factor variables were truncated to boundary (next possible) value. We defined a CpG to be influenced by prenatal factors if the most significant association with the 45 prenatal factor variables had a *P* value < 1 × 10^–3^ (Bonferroni threshold to maintain a family-wise type 1 error rate of 0.05 for testing 45 prenatal factor variables). For each tissue, we report the number and percentage of CpGs whose inter-individual variation could be explained by prenatal factors out of all CpGs that showed inter-individual variation in the tissue. We also report whether the CpG could be explained by prenatal factors in the other tissue. Finally, we contrasted individual EWAS results across 45 prenatal factors for the two infant tissues.

## Results

### Study population

This study used 295 mother–offspring dyads from live singleton term births, with Apgar score ≥ 9, and availability of genotype and DNA methylation data (Additional file 1: Figure A1). Summary statistics of the 295 mother–offspring participants are provided in Additional file 1: Tables A1, 2, and include 49%, 20%, and 30% of subjects from Chinese, Indian, and Malay ethnic groups, respectively. Further, 49% of the neonates were male. We interrogated DNA methylation profiles derived from infant cord tissue and infant cord blood using the Infinium HumanMethylation450 array. After quality control filtering, 239,560 CpGs could be used for subsequent analyses (Additional file 1: Table A3).

### Infant cord tissue DNA methylation showed more inter-individual variability

**Fig. 1:**
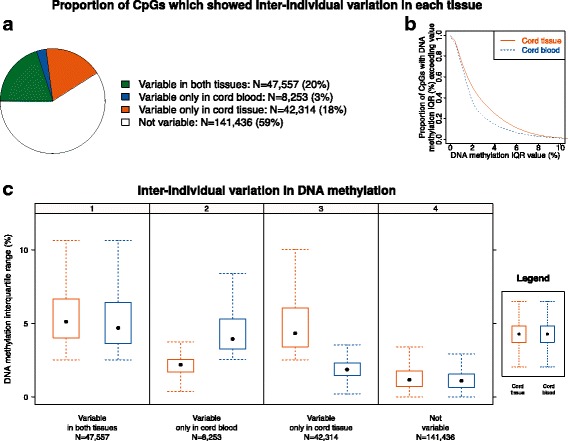
Infant cord tissue showed more inter-individual variation than infant cord blood: proportion of CpGs that showed inter-individual variation and interquartile range (IQR) in DNA methylation. **a** Pie chart shows the proportion of CpGs for four distinct categories: (1) CpGs which showed inter-individual variation in both tissues, (2) CpGs which showed inter-individual variation only in infant cord blood, (3) CpGs which showed inter-individual variation only in infant cord tissue, and (4) CpGs which did not show inter-individual variation in either tissue. A total of 239,560 CpGs passed quality control in both datasets. **b** Plot of proportion of CpGs (vertical axis) in each tissue (out of 239,560 CpGs) with DNA methylation IQR greater than or equal to the value specified on the horizontal axis. **c** Boxplots show the distribution of the DNA methylation IQR, for CpGs in infant cord tissue (bright orange) and infant cord blood (bright blue), respectively, for each of the four categories. Outliers are not shown in the boxplots. A CpG was defined to show inter-individual variation if the DNA methylation range (maximum–minimum, excluding outliers) was greater than 10% and DNA methylation 99th percentile–1st percentile was greater than 5%

As the key focus of an EWAS is to examine the inter-individual variation in DNA methylation, we first characterized and compared the variable CpGs in the two infant tissues (Fig. 1, Additional file 1: Table A4). Of the 239,560 CpGs that passed quality control, 20% exhibited inter-individual variation in both tissues, 21% showed variation in only one dataset, and the remaining 59% did not show variation in any dataset (Fig. 1a). The non-variable CpGs were more likely to be located in promoter regions and CpG islands and were less likely to be in enhancers, while the variable CpGs were more likely to be located in open seas and intronic/intergenic regions and more likely to be in enhancers (Additional file 1: Figures A2–4). Among the tissue-specific CpGs, infant cord tissue had more variable CpGs (18%) (Fig. 1a). In contrast, infant cord blood was less variable, with only 3% of its CpGs exhibiting inter-individual variation specific to cord blood (Fig. 1a). Additionally, as is evident by the interquartile ranges of the CpGs (Fig. 1b, c), the inter-individual variation was higher in the cord tissue than in cord blood. To reduce false positives and to increase statistical power, CpGs that do not exhibit sufficient inter-individual variation are typically excluded from EWAS analysis because their observed variability can potentially be attributed to technical variability [23, 24]. Thus, a point worth noting from this finding is that an EWAS conducted using infant cord tissue would have more CpGs retained for downstream analysis than infant cord blood.

### Neonatal surrogate tissues primarily proxy for tissues/cells of mesodermal origin

**Fig. 2:**
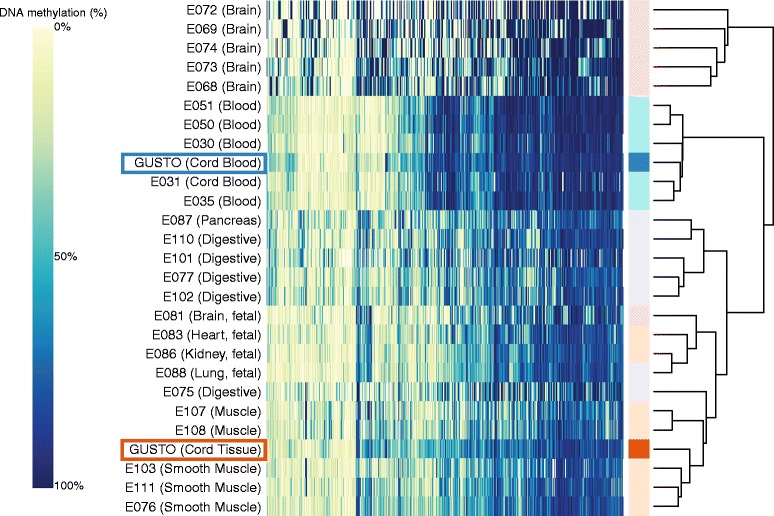
Infant cord tissue is a better surrogate for primary tissues of mesenchymal stem cell (MSC)-derived mesodermic germinal origins, while infant cord blood is a better surrogate for primary tissues of hematopoietic stem cell (HSC)-derived mesodermic germinal origins: hierarchical clustering of GUSTO tissues (cord tissue, cord blood) with 25 primary tissues/cells profiled using reduced representation bisulfite sequencing in the Epigenome Roadmap project. Infant cord tissue clustered more closely with primary tissues of MSC-derived mesodermic germinal origins, while infant cord blood clustered more closely with primary tissues of HSC-derived mesodermic germinal origins. Left panel shows heatmap of DNA methylation values, with each row representing each tissue type and each column representing each CpG. Color changes from yellow to blue as DNA methylation changes from 0% to 100%. Right panel of plot shows dendrogram, with tissue types of ectodermic, endodermic, HSC-derived mesodermic, and MSC-derived mesodermic germinal origins represented in light pink, light purple, light turquoise, and light orange, respectively; GUSTO cord tissue and cord blood are represented in bright orange and bright blue, respectively. DNA methylation values from GUSTO tissues were generated using Infinium 450 K array (for each CpG and tissue type, the median value across all samples was used). For tissues/cells profiled by the Epigenome Roadmap project, only DNA methylation sites with a minimum reads coverage of 30X were retained and reads from both strands were combined. Hierarchical clustering was performed using only CpG sites that passed quality control filtering in GUSTO tissues, were non-missing in at least 10 out of the 25 Epigenome Roadmap samples, and had interquartile range greater than 10% across different Epigenome Roadmap tissues/cells

Infant cord tissue and cord blood are typically used as surrogates for other target tissues [25, 26]. This has strong implications for the clinical relevance of the identified epigenetic signatures as phenotypic biomarkers. To evaluate the similarity of these surrogate tissues with primary tissues, we performed a hierarchical clustering analysis of these infant tissues with 25 primary tissues/cells (Additional file 1: Table B1) profiled using reduced representation bisulfite sequencing under the Epigenome Roadmap project (Fig. 2). These 25 primary tissues/cells comprised a good representation of tissues/cells derived from the ectoderm (e.g., brain, represented in light pink in dendrogram), endoderm (e.g., lung, pancreas, digestive, represented in light purple), mesenchymal stem cell (MSC)-derived mesoderm (e.g., muscle, heart, kidney, represented in light orange), and hematopoietic stem cell (HSC)-derived mesoderm (e.g., blood, represented in light turquoise) germinal origins. Consistent with the findings reported by the Epigenome Roadmap project, tissues/cells generally clustered by their germinal origins (Fig. 2, Additional file 1: Figure B1, Additional file 1: Table B2). Infant cord tissue clustered with MSC-derived mesodermic tissues and fetal tissues, while infant cord blood clustered with the HSC-derived mesodermic tissues (blood).

### Genotype influences a greater proportion of variable CpGs in infant cord blood

**Fig. 3:**
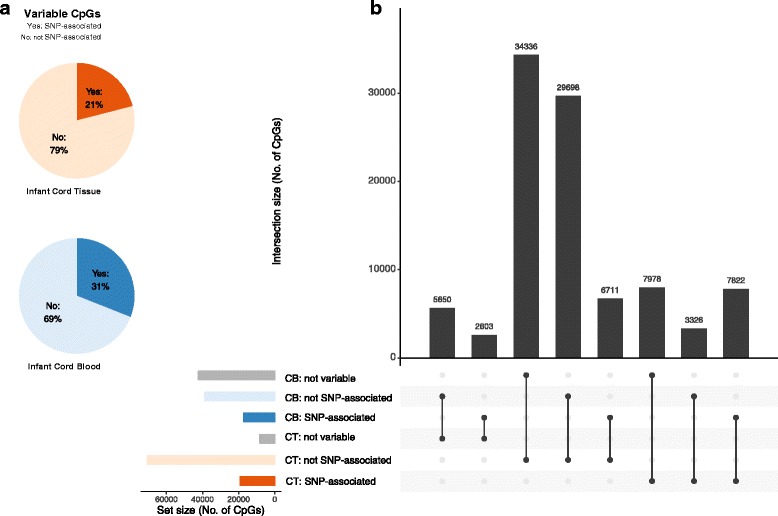
SNPs explained a greater proportion of inter-individual variation in DNA methylation in infant cord blood (CB) than in infant cord tissue (CT): SNP-associated CpGs detected in each infant tissue. **a** Pie charts show the percentage of CpGs in each infant tissue whose inter-individual variation could be explained by SNPs (out of all CpGs which showed inter-individual variation in the infant tissue). A CpG whose inter-individual variation could be explained by SNPs (SNP-associated) was defined to be one where the most significant association between the CpG and *cis*-SNPs (all SNPs on the same chromosome as CpG) attained a *P* value < 5 × 10^–8^, the commonly used Bonferroni threshold for genome-wide association studies (corresponding to testing for 10^6^ independent SNPs across the genome at a family-wise Type 1 error rate of 0.05). **b** Overlap between SNP-associated, non-SNP-associated (but variable), and non-variable CpGs in the two tissues. Only CpGs which showed inter-individual variation in at least one tissue were included (N = 98,124). Examining each tissue separately, each of these 98,124 CpGs can either be SNP-associated, not SNP-associated, or not variable in each tissue. The number of CpGs in each of these three sets in each tissue is shown in the bottom left bar chart (for each tissue the number of CpGs from the three sets will sum to 98,124). Collectively, the 98,124 CpGs can be grouped into eight categories. The bottom right panel identifies each of these eight categories, with the solid black dots representing the sets being considered. For example, the extreme right column identifies the group of CpGs that are SNP-associated in both tissues. The top bar chart shows the number of CpGs in each of these eight categories. For example, 7822 CpGs were SNP-associated in both tissues

We assessed the extent to which genetic variation contributes to inter-individual variability in DNA methylation levels (Fig. 3, Additional file 1: Table C1). Each variable CpG in each infant tissue was regressed against all *cis*-SNPs (SNPs that resided on the same chromosome as the CpG). We found 21% (19,126 CpGs) of the 89,871 variable CpGs in infant cord tissue to be associated with genetic variation (with at least one *cis*-SNP). The corresponding proportion in infant cord blood was 31% (17,136 out of 55,810 CpGs), though infant cord tissue still had more genotype-associated CpGs (19,126 vs. 17,136) due to more variable CpGs. This finding is supported by a previous twin-study which found that genetic factors explained more inter-individual variation in cord blood DNA methylation than in HUVEC DNA methylation [4]. Of note, HUVEC are one of the cell types present in cord tissue. The effect sizes for the CpG-SNP associations in both tissues were similar (Additional file 1: Figure C1). The results from a sensitivity analysis where we adjusted for surrogate variables led to similar conclusions (Additional file 1: Figure C2), though the percentage of SNP-associated CpGs was slightly higher for both tissues (28% for cord tissue and 35% for cord blood).

We further examined the overlap in genotype-associated CpGs from the two tissues (Fig. 3b, Additional file 1: Table C1). The overlap in genotype-associated CpGs between infant cord tissue and cord blood was at 41% or 46%, depending on the number used as denominator. We attempted to replicate the genotype-associated CpGs with those previously reported in cord blood in the Avon Longitudinal Study of Parents and Child (ALSPAC) cohort by Gaunt et al. [2]. Overall, 54% of the genotype-associated CpGs from infant cord blood in our cohort could be replicated in infant cord blood from the ALSPAC cohort (Additional file 1: Table C2). The lack of replication for the remainder of genotype-associated CpGs could be due to ethnic differences in the two cohorts (Asian in GUSTO cohort vs. Caucasian in ALSPAC). Smith et al. [1] reported 131 and 298 genotype-associated CpGs (out of 20,093 CpGs analyzed) in African American and Caucasian infant cord blood, respectively, with a similar degree of overlap between African American and Caucasian infant cord blood (96 CpGs, 32% or 73% depending on the number used as the denominator).

### EWAS associations in the two surrogate tissues were distinct

**Fig. 4:**
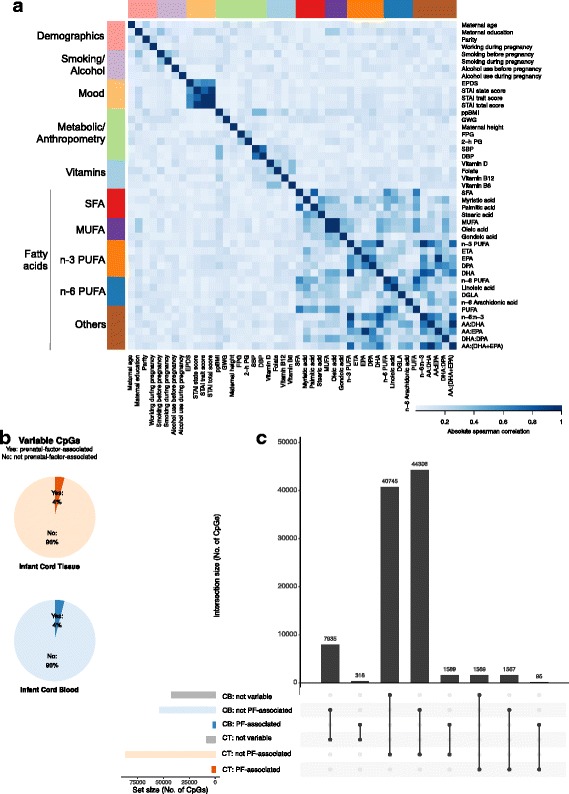
Prenatal factors (PFs) explained a similar proportion of inter-individual variation in infant cord blood (CB) and infant cord tissue (CT): CpGs where the inter-individual variation in DNA methylation were explained by PFs. **a** Heatmap shows the pairwise Spearman correlation (absolute value) between 45 PFs. Each row/column represents each PF. Color changes from white to blue as correlation changes from zero to one. **b** Pie charts show the percentage of CpGs in each infant tissue whose inter-individual variation could be explained by PFs (out of all CpGs, which showed inter-individual variation in the infant tissue). A CpG whose inter-individual variation could be explained by PFs was defined to be one where the most significant association between the CpG and all 45 PFs attained a *P* value < 1 × 10^–3^, the Bonferroni threshold for testing 45 PFs at a family-wise Type 1 error rate of 0.05. **c** Overlap between PF-associated, non-PF-associated (but variable), and non-variable CpGs in the two tissues. Only CpGs which showed inter-individual variation in at least one tissue were included (N = 98,124). Examining each tissue separately, each of these 98,124 CpGs can either be PF-associated, non-PF-associated, or not variable in each tissue. The number of CpGs in each of these three sets in each tissue is shown in the bottom left bar chart. Collectively, the 98,124 CpGs can be grouped into eight categories. The bottom right panel identifies each of these eight categories, with the solid black dots representing the sets being considered. The top bar chart shows the number of CpGs in each of these eight categories

We investigated the role of prenatal factors in contributing to the inter-individual variability in DNA methylation levels. For this, we regressed all variable CpGs in each tissue with 45 prenatal factor variables separately. A list of these 45 variables and their pairwise correlation is shown in Fig. 4a. A CpG was defined to be associated with the prenatal factors if it was associated with at least 1 of the 45 prenatal factor variables. As an overall characterization of the DNA methylation–prenatal factor relationship, we computed the number/percentage of variable CpGs that were associated with the prenatal factors in each tissue (Fig. 4b), and the overlap in prenatal factor-associated CpGs in the two tissues (Fig. 4c). The prenatal factors as a whole explained a similar proportion (4% of variable CpGs) of inter-individual variation in both tissues (Fig. 4b). The overlap in prenatal factor-associated CpGs in the two tissues was low (Fig. 4b, Additional file 1: Table D1). The DNA methylation–prenatal factor effect sizes in both tissues were similar (Additional file 1: Figure D1). A subset of these prenatal factor-associated CpGs also showed association with genetic variation, at 22% and 32% for infant cord tissue and infant cord blood, respectively (Additional file 1: Table D2). In both the surrogate tissues, CpGs associated or not with prenatal factors showed similar inter-individual variation and a similar distribution of genomic features. Prenatal factor-associated CpGs in both the tissues did not show significant enrichment in any gene ontology pathways. Finally, we also contrasted the effects of individual prenatal factors on individual CpGs in the infant tissues (Additional file 1: Figures D2, 3). For a CpG associated with a prenatal factor in infant cord tissue, we examined if this CpG was also associated with the same prenatal factor in infant cord blood (Additional file 1: Figure D2). We also attempted the reverse analyses (Additional file 1: Figure D3). In general, we noticed a low concordance in EWAS results from the two neonatal tissues. Sensitivity analysis, where we adjusted for surrogate variables, led to similar conclusions (Additional file 1: Figures D4–6).

## Discussion

This study reports a comprehensive analysis of inter-individual variation in genome-wide DNA methylation in two routinely collected surrogate tissues at birth (cord tissue and cord blood). This comparison between infant cord tissue and cord blood highlights the importance of considering tissue specificity in interrogating the relationship between genetic and prenatal factors with epigenetic variation in neonatal tissues. Our findings suggest that, for a neonatal EWAS conducted using the cord tissue versus the cord blood, there will be (1) more variable CpGs retained for subsequent phenotype association analysis, (2) these variable CpGs will be less likely to be associated with genotype, (3) but equally likely to be associated with prenatal factors, and finally, (4) cord tissue will serve as a better surrogate for target tissues of MSC origin.

Our tissue-specific findings provide better insights into tissue selection and hypotheses that can be addressed in future neonatal EWAS. For discovery-based studies related to a phenotype of interest, examining more than one surrogate tissue can provide a more comprehensive understanding of the underlying biological mechanisms, and the future potential of the surrogate tissues in a clinical setting. EWAS in surrogate tissue can also be used for the purpose of identifying biomarkers, though the identified biomarkers need not always reflect the underlying biological mechanisms in the primary tissues, and might be surrogate tissue specific. Since it is quite likely that contrasting results will be obtained when comparing EWAS findings from different neonatal tissues, our findings also imply being careful when attempting replication analyses, as it will be more reproducible when conducted on the same tissue type. Likewise, replication studies attempted on different tissue types should be carefully interpreted and the caveats discussed accordingly.

Findings from this study also provide evidence for the utility of infant cord tissue in a neonatal EWAS. To date, large sample size EWAS (N > 100) have primarily been attempted on infant cord blood. This study demonstrates that infant cord tissue can capture distinct DNA methylation signatures and prenatal factor influences from infant cord blood. Additionally, the closer clustering of cord tissue with the MSC-derived mesodermic tissues, such as skeletal muscle and smooth muscle, suggests that cord tissue is a better surrogate for these primary tissues than cord blood, although this would require further experimental validation in future studies.

This study has some limitations. First, even though we adjusted for cellular heterogeneity using a reference panel, residual confounding effects could persist. In such a scenario, DNA methylation–prenatal factor associations will be more susceptible to these effects than the DNA methylation–genotype associations. Developing better reference panels will alleviate such limitations. Second, the higher number of variable CpGs in cord tissue could arise due to increased diversity of cell types in cord tissue. For example, cord tissue probably consists of a mixture of stromal, endothelial, epithelial, and blood contamination [27], while cord blood consists of different leukocytes. To adequately interrogate this possibility, future studies will require fractionating constituent cell types of cord tissue and cord blood and comparing their DNA methylation profiles. Third, we did not profile DNA methylation from placenta or buccal cells at birth, or additional tissues later in the life-course. Examination of the buccal DNA methylome could be useful at subsequent stages of child growth as buccal samples are non-invasive and more accessible, thus enabling comparison of DNA methylation patterns across the life-course. Additionally, studies have reported that buccal cells might be a better surrogate for brain tissue than blood [28]. On the other hand, placenta and cord tissue can only be examined for neonatal EWAS. Further, while cord blood DNA methylation can be compared to DNA methylation patterns in blood taken at later stages in life-course, blood samples are typically not available in early childhood. As of now, it remains unclear how EWAS findings from cord tissue and cord blood would relate to those from placenta, buccal cells, or tissues derived later in the life-course. Future research is necessary to address this question. Finally, while our study sample size (N = 295) is larger than most sample sizes used in previous tissue-specificity investigations (N < 50–100), our study could still be underpowered, especially in the examination of DNA methylation–prenatal factor associations with small effect sizes. In examining the DNA methylation–prenatal factor associations, we have used a less conservative threshold of 1 × 10^–3^. However, our study sample size is comparable to frequently utilized sample sizes for most of the prenatal factors interrogated in this study. Thus, our observations from neonate EWAS contributes to the current understanding of prenatal factor influences on the fetus in utero.

## Conclusion

There has been a considerable increase in the use of EWAS analysis to study the developmental origins of health and disease. However, it is becoming increasingly evident that EWAS studies are more complicated than genome-wide association studies as epigenetic markers are dynamic, tissue specific, and influenced by genetic and environmental factors. Thus, designing an EWAS warrants multiple considerations to facilitate the identification of a reliable epigenetic signal, especially from the surrogate tissues. This study emphasizes that the epigenetic-genetic-prenatal factor relationship is tissue specific and the choice of neonatal tissues used for EWAS analyses is important to enhance the scope and replication of the epigenetic findings in future studies.

## Additional files

Additional file 1: Supplementary tables and figures. (PDF 2720 kb)

Additional file 2: Tab-delimited file containing DNA methylation values for 295 cord tissue samples, 239,560 CpGs. (TXT 615004 kb)

Additional file 3: Tab-delimited file containing DNA methylation values for 295 cord blood samples, 239,560 CpGs. (TXT 613902 kb)

Additional file 4: Information on using DNA methylation data in Additional files 2 and 3. (PDF 36 kb)

## Abbreviations

AA: Arachidonic acid
ALSPAC: Avon longitudinal study of parents and child
DBP: Diastolic blood pressure
DGLA: Dihomo-gamma-linolenic acid
DHA: Docosahexaenoic acid
DPA: Docosapentaenoic acid
ENCODE: Encyclopedia of DNA Elements
EPA: Eicosapentaenoic acid
EPDS: Edinburgh postnatal depression scale
ETA: Eicosatetraenoic acid
EWAS: Epigenome wide association studies
FANTOM: Functional Annotation of the Mammalian genome
FPG: Fasting plasma glucose
GA: Gestational age
GWG: Gestational weight gain
GUSTO: Growing Up in Singapore Towards Healthy Outcomes
HSC: Hematopoietic stem cell
HUVEC: Human umbilical vein endothelial cells
KKH: KK Women’s and Children’s Hospital, Singapore
MSC: Mesenchymal stem cell
MUFA: Monounsaturated fatty acid
NUH: National University Hospital, Singapore
PCA: Principal component analysis
PUFA: Polyunsaturated fatty acid
ppBMI: Pre-pregnancy BMI
SBP: Systolic blood pressure
SFA: Saturated fatty acid
SNP: Single Nucleotide Polymorphism
STAI: State-trait anxiety inventory
2-h PG: 2-hr post-glucose
