# Supplementary material for: Choice of surrogate tissue influences neonatal EWAS findings
Source: BMC Med. 2017 Dec 5;15:211. doi: 10.1186/s12916-017-0970-x (PMC5715509; doi:10.1186/s12916-017-0970-x)

# Choice of surrogate tissue influences neonatal EWAS findings

Supplementary Material

Hyperlink

# Contents


**Supplementary A: Summary statistics, data QC & genomic annotation** 2

**Supplementary B: Clustering with Epigenome Roadmap data** 9

**Supplementary C: G model summary** 13

**Supplementary D: E model summary** 17


Hyperlink

Supplementary Figure A1: **Study inclusion/exclusion criteria.**

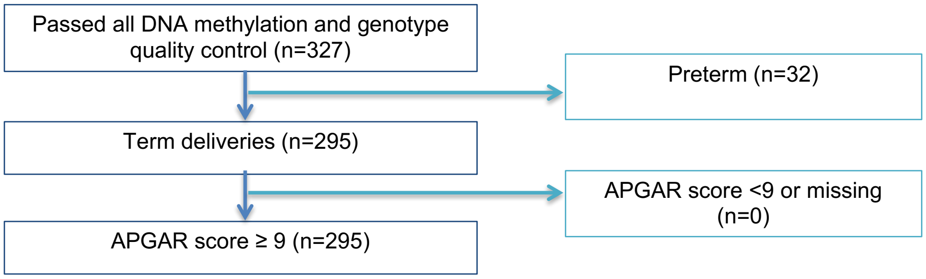

**Supplementary Table A1:** Maternal characteristics of the GUSTO cohort studied in the analysis.

| | Time point | N (%) | Mean (SD) |
|---|---|---|---|
| **Maternal mood** | | | |
| EPDS score | 26-28 weeks gestation | 283 | 8.1 (4.5) |
| STAI state score | | 285 | 34.8 (10.3) |
| STAI trait score | | 285 | 36.3 (10.3) |
| STAI total score | | 285 | 71.1 (19.4) |
| **Metabolic/Anthropometry** | | | |
| Pre-pregnancy BMI (kg/m$^2$) | Self-reported at first clinic visit | 269 | 23.3 (4.9) |
| Gestational weight gain (kg) | 26-28 weeks gestation | 267 | 8.5 (5.2) |
| Maternal height (cm) | | 290 | 157.7 (5.8) |
| Fasting plasma glucose (mmol/l) | | 276 | 4.4 (0.4) |
| 2hr plasma glucose (mmol/l) | | 276 | 6.5 (1.7) |
| Systolic blood pressure (mm Hg) | | 261 | 112.3 (11.5) |
| Diastolic blood pressure (mm Hg) | | 261 | 64.3 (7.3) |
| **Saturated fatty acids (SFA)** | | | |
| SFA (%) | 26-28 weeks gestation | 286 | 45.7 (3.2) |
| Myristic acid (%) | | 286 | 0.5 (0.2) |
| Palmitic acid (%) | | 286 | 34.0 (3.5) |
| Stearic acid (%) | | 286 | 11.1 (1.7) |
| **Monounsaturated fatty acids (MUFA)** | | | |
| MUFA (%) | 26-28 weeks gestation | 286 | 13.7 (2.3) |
| Oleic acid (%) | | 286 | 11.2 (2.0) |
| Gondoic acid (%) | | 286 | 0.2 (0.2) |
| **n-3 Polyunsaturated fatty acids (n-3 PUFA)** | | | |
| n-3 PUFA (%) | 26-28 weeks gestation | 286 | 6.3 (1.7) |
| Eicosatetraenoic acid (ETA) (%) | | 286 | 0.2 (0.2) |
| EPA (%) | | 286 | 0.7 (0.5) |
| DPA (%) | | 286 | 0.6 (0.2) |
| DHA (%) | | 286 | 4.7 (1.3) |
| **n-6 Polyunsaturated fatty acids (n-6 PUFA)** | | | |
| n-6 PUFA (%) | 26-28 weeks gestation | 286 | 34.3 (3.3) |
| Linoleic acid (%) | | 286 | 21.7 (3.4) |
| Dihomo-gamma-linolenic acid (DGLA) (%) | | 285 | 4.0 (1.3) |
| n-6 Arachidonic acid (%) | | 286 | 7.9 (1.8) |
| **Others** | | | |
| PUFA (%) | 26-28 weeks gestation | 286 | 40.6 (3.4) |
| Ratio of n-6:n-3 | | 286 | 5.8 (1.8) |
| Ratio of AA:DHA | | 286 | 1.8 (0.6) |
| Ratio of AA:EPA | | 286 | 17.5 (11.2) |
| Ratio of DHA:DPA | | 286 | 8.4 (3.1) |
| Ratio of AA:(DHA+EPA) | | 286 | 1.6 (0.5) |

**Supplementary Table A2:** Maternal characteristics of the GUSTO cohort studied in the analysis.

| | | Time point | N (%) | Mean (SD) |
|---|---|---|---|---|
| **Demographics** | | | | |
| Maternal age | ≥ 35y | Self-reported at first clinic visit | 68 (23%) | |
| | < 35y | | 227 (77%) | |
| Maternal education | ≥ 12y | | 160 (55%) | |
| | < 12y | | 132 (45%) | |
| Parity | > 0 | Delivery | 164 (56%) | |
| | 0 | | 131 (44%) | |
| Working during pregnancy | Yes | Interviewer-administered questionnaire at 26-28 weeks gestation | 187 (64%) | |
| | No | | 106 (36%) | |
| **Smoking/Alcohol** | | | | |
| Smoking before pregnancy | Yes | Interviewer-administered questionnaire at 26-28 weeks gestation | 41 (14%) | |
| | No | | 251 (86%) | |
| Smoking during pregnancy | Yes | | 6 (2%) | |
| | No | | 287 (98%) | |
| Alcohol use before pregnancy | Yes | | 95 (32%) | |
| | No | | 198 (68%) | |
| Alcohol use during pregnancy | Yes | | 6 (2%) | |
| | No | | 282 (98%) | |
| **Vitamins** | | | | |
| Plasma vitamin D | > 50 nmol/l | 26-28 weeks gestation | 233 (85%) | |
| | ≤ 50 nmol/l | | 40 (15%) | |
| Plasma folate | ≥ 6 ng/ml | | 254 (88%) | |
| | < 6 ng/ml | | 34 (12%) | |
| Plasma vitamin B12 | ≥ 300 pg/ml | | 111 (39%) | |
| | < 300 pg/ml | | 177 (61%) | |
| Plasma vitamin B6 | < 20 nmol/l | | 39 (14%) | |
| | ≥ 20 nmol/l | | 248 (86%) | |

Supplementary Table A3: **Number of CpGs that passed all quality control filtering in each of the two DNA methylation datasets (infant cord tissue, infant cord blood).** This analysis used 295 samples that had both infant cord tissue and infant cord blood DNA methylation data. The cord tissue DNA methylation dataset was part of a larger dataset that included other samples and a CpG was considered to pass quality control filtering only if it passed quality control filtering in the larger dataset. Likewise, the cord blood DNA methylation dataset was part of a larger dataset that included other samples, and a CpG was considered to pass quality control filtering only if it passed quality control filtering in the larger dataset. We retained only CpGs that had non-missingness in all the samples (larger dataset) assayed.

| | No. of CpGs |
|---:|---:|
| Passed QC in infant cord tissue | 262,891 |
| Passed QC in infant cord blood | 351,868 |
| Passed QC in both datasets | 239,560 |

Supplementary Table A4: **Final number of CpGs that showed inter-individual variation in each of the two DNA methylation datasets (infant cord tissue, infant cord blood).**

| | No. of CpGs |
|---:|---:|
| Variable in infant cord tissue | 89,871 |
| Variable in infant cord blood | 55,810 |
| Variable in both datasets | 47,557 |

Supplementary Figure A2: **CpGs which showed inter-individual variation in either tissue were more likely to be located in open seas while CpGs which did not show inter-individual variation in both tissues were more likely to be located in CpG islands: CpG content distribution (island, shore, shelf and open sea) for four different groups of CpGs.** Epigenome-wide CpGs that passed quality control in both tissues ($N = 239,560$) were segregated into four distinct categories: (1) CpGs which showed inter-individual variation in both tissues, (2) CpGs which showed inter-individual variation only in infant cord blood, (3) CpGs which showed inter-individual variation only in infant cord tissue and (4) CpGs which did not show inter-individual variation in both tissues. Each group of CpGs were annotated in terms of their CpG content distribution.

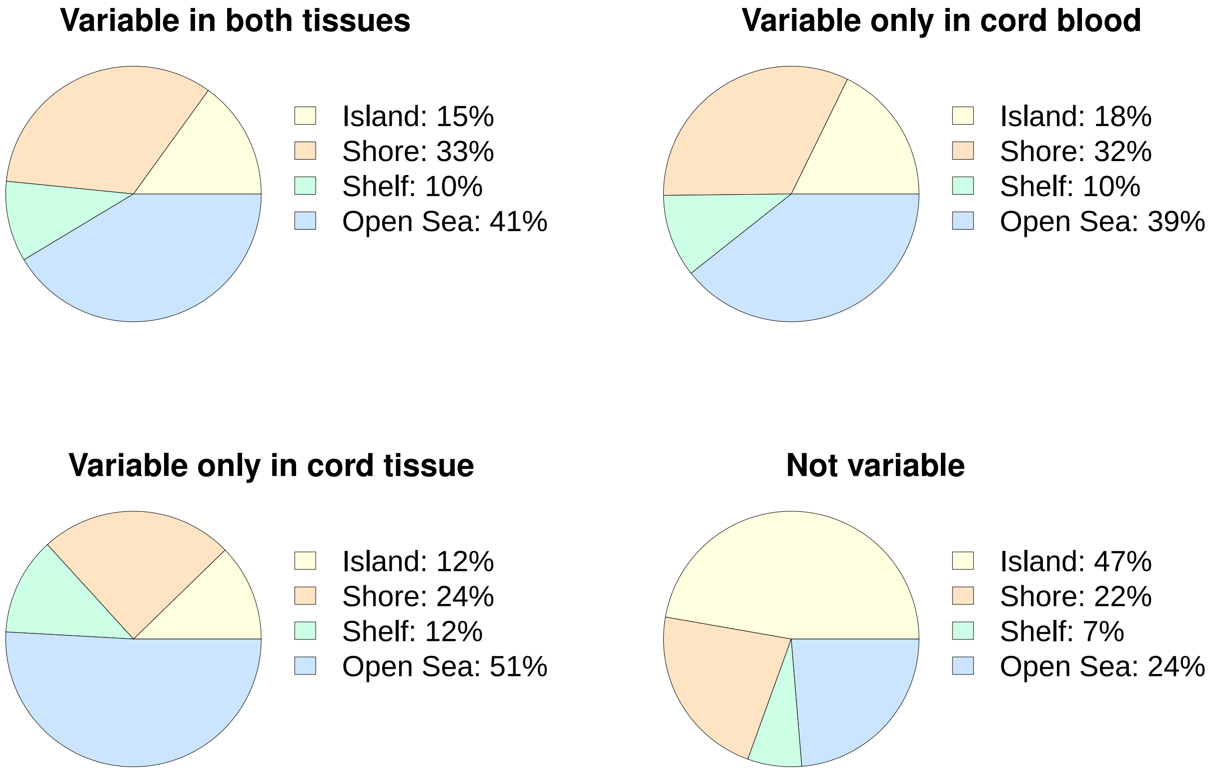

Supplementary Figure A3: **CpGs which showed inter-individual variation in either tissue were more likely to be in intronic/intergenic regions while CpGs which did not show inter-individual variation in both tissues were more likely to be in promoter regions: Functional genomic distribution (promoter, 5'-UTR, exon, intron, 3'UTR, TTS and intergenic) for four different groups of CpGs.** Epigenome-wide CpGs that passed quality control in both tissues ($N = 239,560$) were segregated into four distinct categories: (1) CpGs which showed inter-individual variation in both tissues, (2) CpGs which showed inter-individual variation only in infant cord blood, (3) CpGs which showed inter-individual variation only in infant cord tissue and (4) CpGs which did not show inter-individual variation in both tissues. Each group of CpGs were annotated in terms of their functional genomic distribution using Homer.

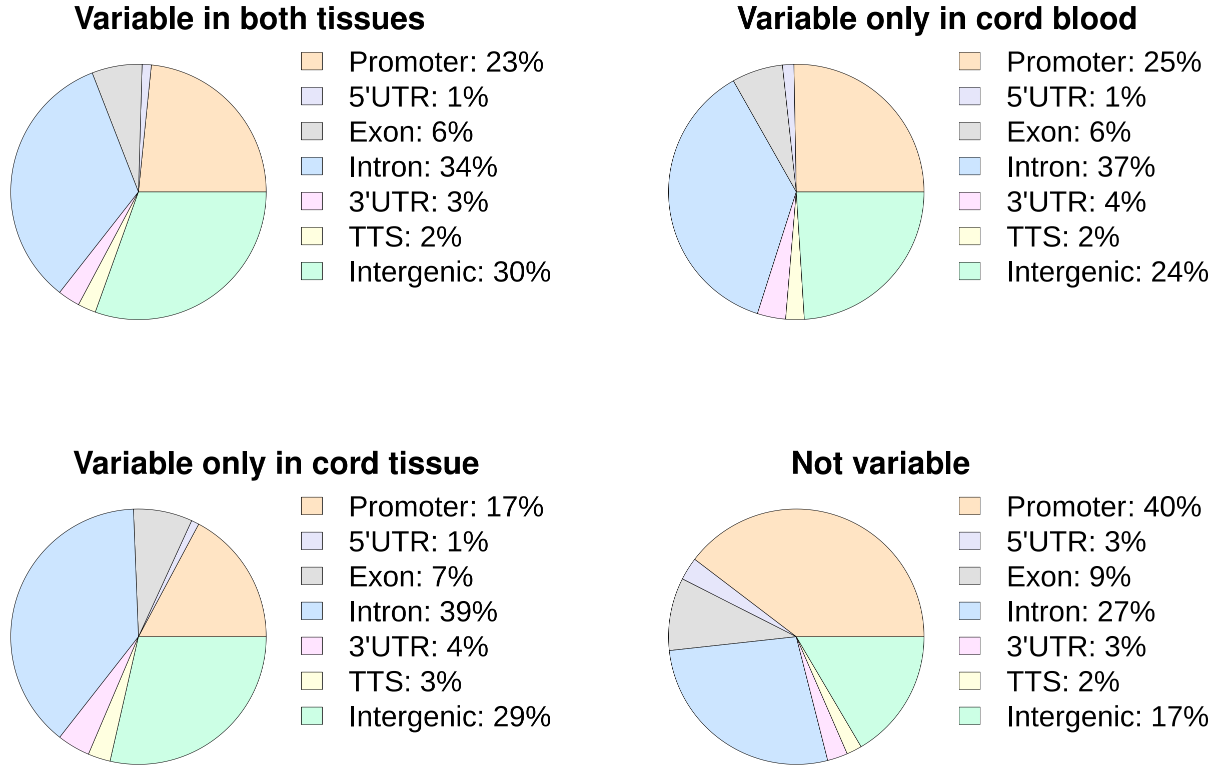

Supplementary Figure A4: **CpGs which showed inter-individual variation in either tissue were more likely to be predicted enhancers while CpGs which did not show inter-individual variation in both tissues were less likely to be predicted enhancers: Percentage of CpGs which were classified as predicted enhancers for four different groups of CpGs.** Epigenome-wide CpGs that passed quality control in both tissues ($N = 239,560$) were segregated into four distinct categories: (1) CpGs which showed inter-individual variation in both tissues, (2) CpGs which showed inter-individual variation only in infant cord blood, (3) CpGs which showed inter-individual variation only in infant cord tissue and (4) CpGs which did not show inter-individual variation in both tissues. Each group of CpGs were annotated in terms of whether they were predicted enhancers by either the Encyclopedia of DNA Elements (ENCODE) consortium or The FANTOM (Functional Annotation of the Mammalian Genome) consortium. For predicted enhancers from ENCODE, we used the annotation that was included in the Infinium HumanMethylation450 manifest file. The FANTOM5 predicted enhancer annotation was obtained by using FANTOM5 Phase 1 and Phase 2 data.

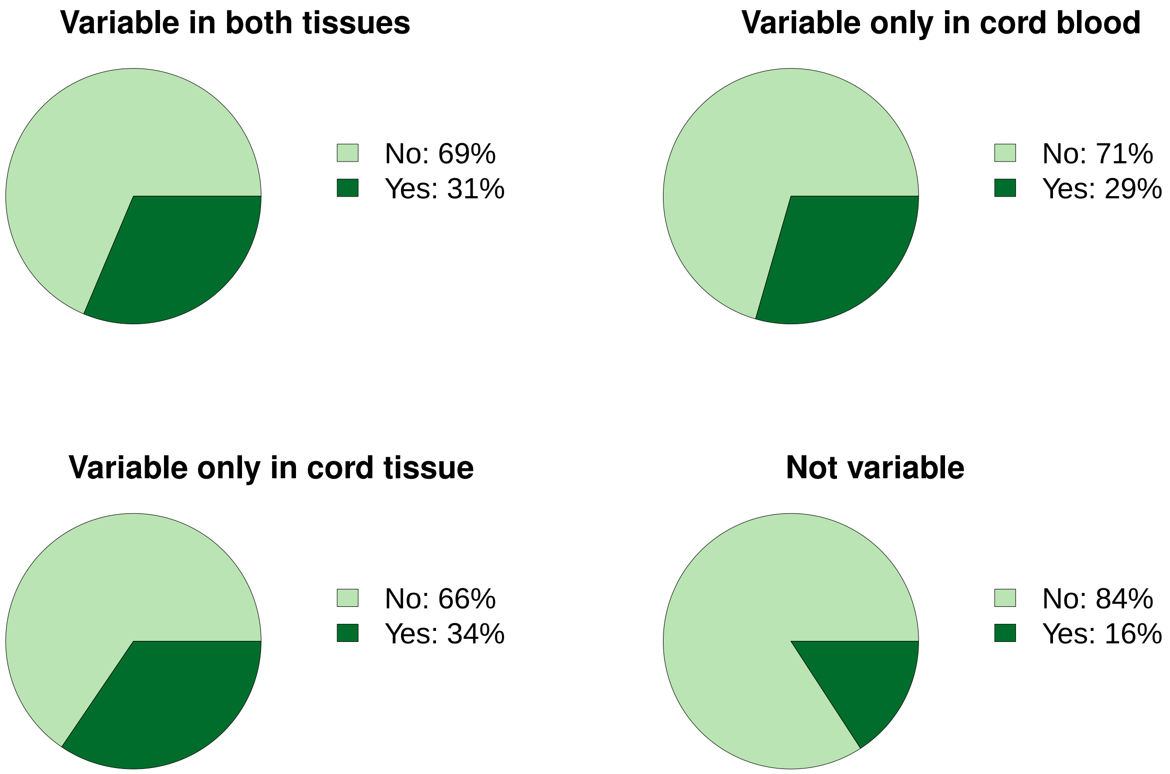

Supplementary Table B1: **List of primary tissues/cells profiled using reduced representation bisulfite sequencing (RRBS) in the Epigenome Roadmap project.**

| Cell type/tissue group | EID | Epigenome name |
|---|---|---|
| Blood | E030 | Primary neutrophils (from PB) |
| Blood | E031 | Primary B cells from cord blood |
| Blood | E035 | Primary haematopoietic stem cells (HSCs) |
| Blood | E050 | Primary HSCs G-CSF-mobilized female |
| Blood | E051 | Primary HSCs G-CSF-mobilized male |
| Brain | E068 | Brain anterior caudate |
| Brain | E069 | Brain cingulate gyrus |
| Brain | E072 | Brain inferior temporal lobe |
| Brain | E073 | Brain dorsolateral prefontal cortex |
| Brain | E074 | Brain substantia nigra |
| Digestive | E075 | Colonic mucosa |
| Smooth Muscle | E076 | Colon smooth muscle |
| Digestive | E077 | Duodenum mucosa |
| Brain, fetal | E081 | Fetal brain male |
| Heart, fetal | E083 | Fetal heart |
| Kidney, fetal | E086 | Fetal kidney |
| Pancreas | E087 | Pancreatic islets |
| Lung, fetal | E088 | Fetal lung |
| Digestive | E101 | Rectal mucosa donor 29 |
| Digestive | E102 | Rectal mucosa donor 31 |
| Smooth Muscle | E103 | Rectal smooth muscle |
| Muscle | E107 | Skeletal muscle male |
| Muscle | E108 | Skeletal muscle female |
| Digestive | E110 | Stomach mucosa |
| Smooth Muscle | E111 | Stomach smooth muscle |

Supplementary Figure B1: **Correlation in DNA methylation values between GUSTO tissues (cord tissue, cord blood) and primary tissues/cells profiled using RRBS from Epigenome Roadmap project.** Left panel shows a heatmap, where the color represents the spearman correlation between each GUSTO tissue and each of the primary tissues/cells profiled using RRBS from Epigenome Roadmap project. Each column represents each GUSTO tissue type and each row represents each Epigenome Roadmap primary tissue/cell. Color changes from yellow to red as spearman correlation increases. DNA methylation values from GUSTO tissues were generated using Infinium 450K array. Only CpG sites which passed quality control filtering ($N = 239,560$) in the GUSTO tissues (cord tissue, cord blood) were used in the computation of the spearman correlation (extreme DNA methylation values in GUSTO tissues were further excluded from the computation). DNA methylation values of 25 tissue types were generated using reduced representation bisulfite sequencing (RRBS) by the Epigenome Roadmap project. Only DNA methylation sites with a minimum reads coverage of 30X were retained and reads from both strands were combined. For each GUSTO tissue type, the median (across 295 individuals) spearman correlation is shown. Right panel shows a dendrogram, which was generated independently of the heatmap (left panel). Tissue types of ectodermic, endodermic, mesodermic (HSC-derived) and mesodermic (MSC-derived) germinal origins are shown in light pink, light purple, light turquoise and light orange, respectively. Hierarchical clustering was performed on the 25 primary tissues/cells profiled using RRBS from Epigenome Roadmap project (without GUSTO tissues), using DNA methylation sites that were non-missing in at least 10 out of the 25 Epigenome Roadmap samples and that had interquartile range $> 10\%$ across different Epigenome Roadmap tissues/cells. Hierarchical clustering was performed using the Ward's minimum variance method and euclidean distance.

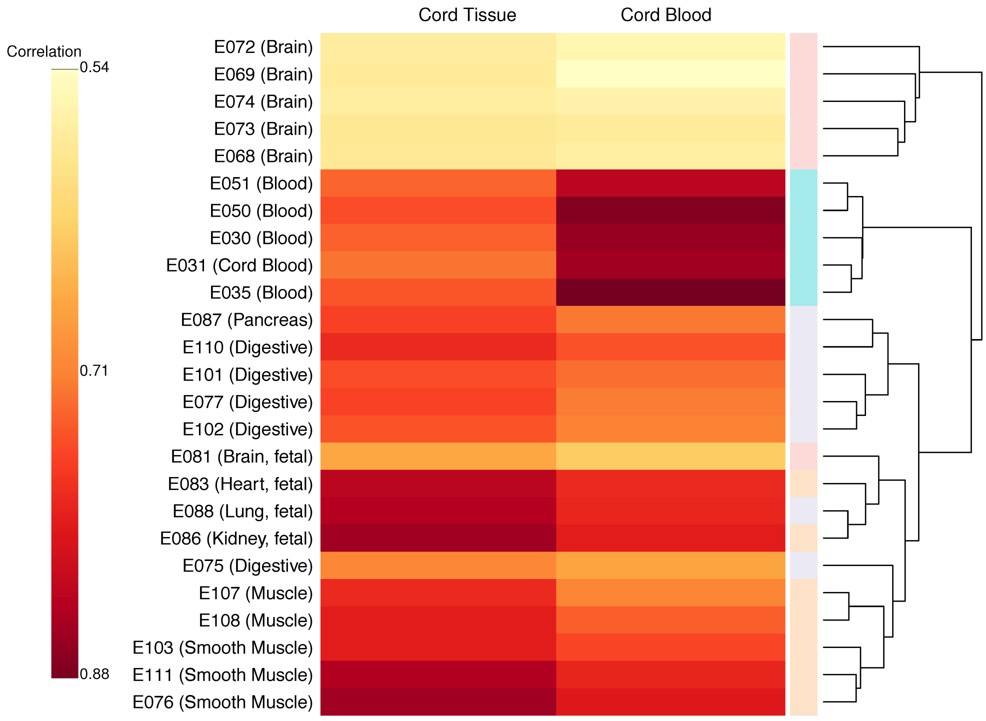


Hyperlink

Supplementary Table B2: **Correlation in DNA methylation values between GUSTO tissues (cord tissue, cord blood) and primary tissues/cells profiled using RRBS from Epigenome Roadmap project.** We computed the spearman correlation between each GUSTO tissue and each of the primary tissues/cells profiled using RRBS from Epigenome Roadmap project. Each column represents each GUSTO tissue type and each row represents each Epigenome Roadmap primary tissue/cell. DNA methylation values from GUSTO tissues were generated using Infinium 450K array. Only CpG sites which passed quality control filtering ($N = 239,560$) in the GUSTO tissues (cord tissue, cord blood) were used in the computation of the spearman correlation (extreme DNA methylation values in GUSTO tissues were further excluded from the computation). DNA methylation values of 25 tissue types were generated using reduced representation bisulfite sequencing (RRBS) by the Epigenome Roadmap project. Only DNA methylation sites with a minimum reads coverage of 30X were retained and reads from both strands were combined. For each GUSTO tissue type, the median (across 295 individuals) spearman correlation is shown. The spearman correlation values are also represented in the left panel of Supplementary Figure B1.

| | Infant Cord Tissue | Infant Cord Blood |
|---|---|---|
| E072 (Brain) | 0.58 | 0.56 |
| E069 (Brain) | 0.58 | 0.54 |
| E074 (Brain) | 0.57 | 0.57 |
| E073 (Brain) | 0.59 | 0.58 |
| E068 (Brain) | 0.58 | 0.57 |
| E051 (Blood) | 0.73 | 0.83 |
| E050 (Blood) | 0.75 | 0.87 |
| E030 (Blood) | 0.73 | 0.85 |
| E031 (Cord Blood) | 0.72 | 0.85 |
| E035 (Blood) | 0.74 | 0.88 |
| E087 (Pancreas) | 0.75 | 0.71 |
| E110 (Digestive) | 0.78 | 0.74 |
| E101 (Digestive) | 0.74 | 0.72 |
| E077 (Digestive) | 0.75 | 0.71 |
| E102 (Digestive) | 0.74 | 0.71 |
| E081 (Brain, fetal) | 0.67 | 0.63 |
| E083 (Heart, fetal) | 0.83 | 0.77 |
| E088 (Lung, fetal) | 0.83 | 0.78 |
| E086 (Kidney, fetal) | 0.85 | 0.79 |
| E075 (Digestive) | 0.70 | 0.67 |
| E107 (Muscle) | 0.77 | 0.71 |
| E108 (Muscle) | 0.79 | 0.73 |
| E103 (Smooth Muscle) | 0.79 | 0.75 |
| E111 (Smooth Muscle) | 0.84 | 0.78 |
| E076 (Smooth Muscle) | 0.85 | 0.79 |

Supplementary Figure B2: **Hierarchical clustering of GUSTO tissues (cord tissue, cord blood).** DNA methylation values from GUSTO tissues were generated using Infinium 450K array. Hierarchical clustering was performed using all CpG sites which passed quality control filtering ($N = 239,560$) in the two GUSTO tissues (cord tissue, cord blood). All 295 infants clustered by cell type (data not shown). Here the clustering results are shown for eighteen infants.

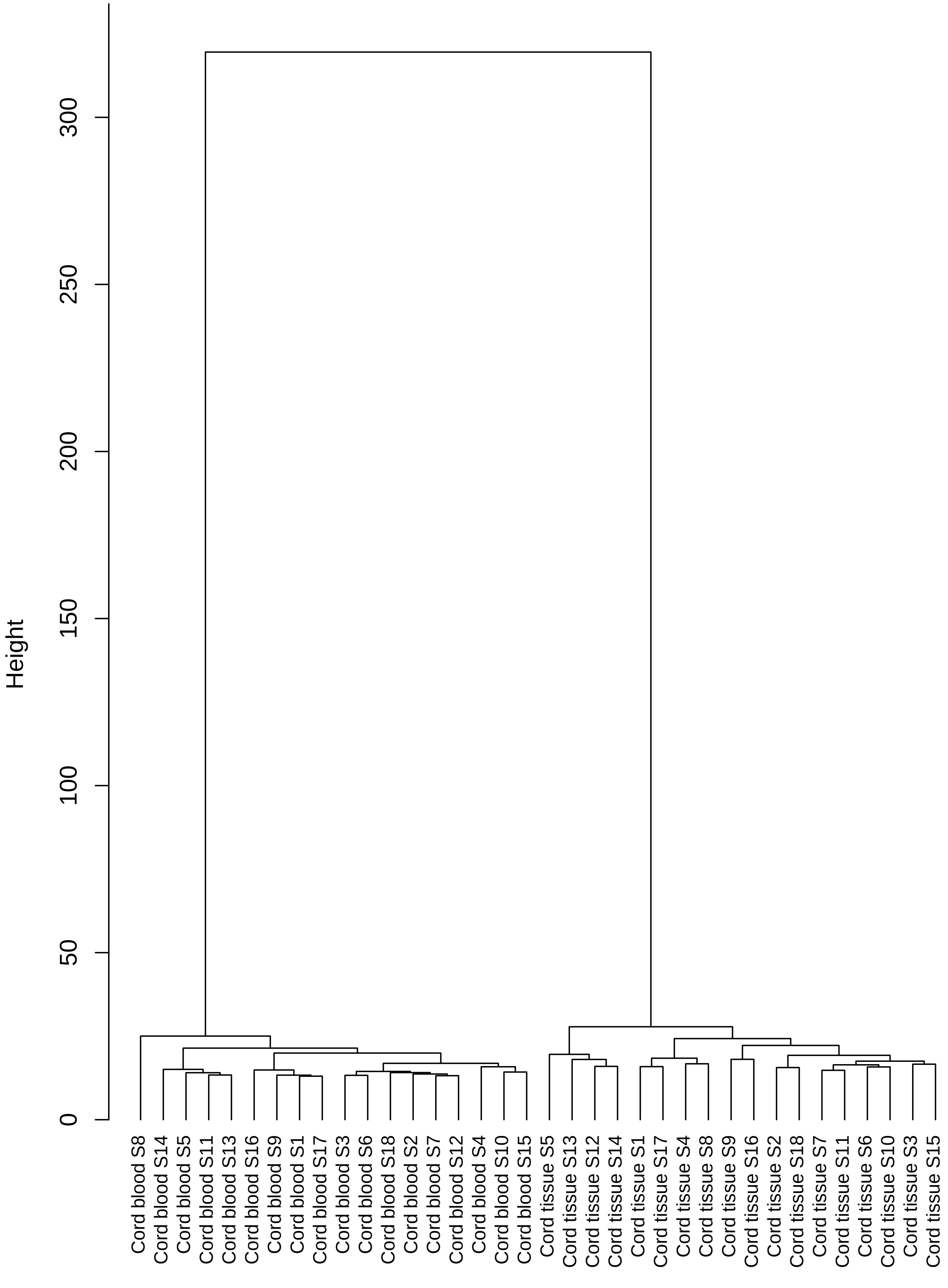


Hyperlink

Supplementary Table C1: **SNP-associated CpGs detected in each infant tissue.** Table shows the number of SNP-associated CpGs detected in each infant tissue, and whether the CpG was SNP-associated in the other infant tissue. A CpG was defined to be SNP-associated if the most significant association between the CpG and *cis*-SNPs (all SNPs on the same chromosome as CpG) attained a p-value $< 5 \times 10^{-8}$, the commonly used bonferroni threshold for genome-wide association studies (corresponding to testing for $\sim 10^6$ independent SNPs across the genome at a family-wise Type 1 error rate of 0.05).

SNP-associated CpGs in Infant Cord Tissue

| | Infant Cord Blood |
|---|---|
| SNP-associated | 7822 (41%) |
| Not SNP-associated | 3326 (17%) |
| Not variable | 7978 (42%) |
| Total | 19126 (100%) |

SNP-associated CpGs in Infant Cord Blood

| | Infant Cord Tissue |
|---|---|
| SNP-associated | 7822 (46%) |
| Not SNP-associated | 6711 (39%) |
| Not variable | 2603 (15%) |
| Total | 17136 (100%) |

Supplementary Figure C1: **Effect sizes for CpG-SNP associations in each infant tissue.** Volcano plots (top panel) shows the effect-sizes of CpG-SNP associations for all variable CpGs in each infant tissue. Vertical axis shows the $-\log_{10}(\text{p-value})$ for CpG-SNP association and horizontal axis shows the CpG-SNP effect sizes. Density of blue color represents the density of points/CpGs (darker blue represents higher density of points/CpGs). SNP-associated CpGs are also represented as black points. Effect sizes are represented as change in DNA methylation Z-score for each additional allele of SNP. DNA methylation Z-score for each CpG was computed by scaling DNA methylation values to mean zero and unit variance in each tissue separately. Boxplot (bottom panel) gives the (absolute values of) effect-sizes of CpG-SNP associations only for SNP-associated CpGs in each tissue. Outliers are not shown in the boxplots. A CpG was defined to be SNP-associated if the most significant association between the CpG and *cis*-SNPs (all SNPs on the same chromosome as CpG) attained a p-value $< 5 \times 10^{-8}$, the commonly used bonferroni threshold for genome-wide association studies (corresponding to testing for $\sim 10^6$ independent SNPs across the genome at a family-wise Type 1 error rate of 0.05).

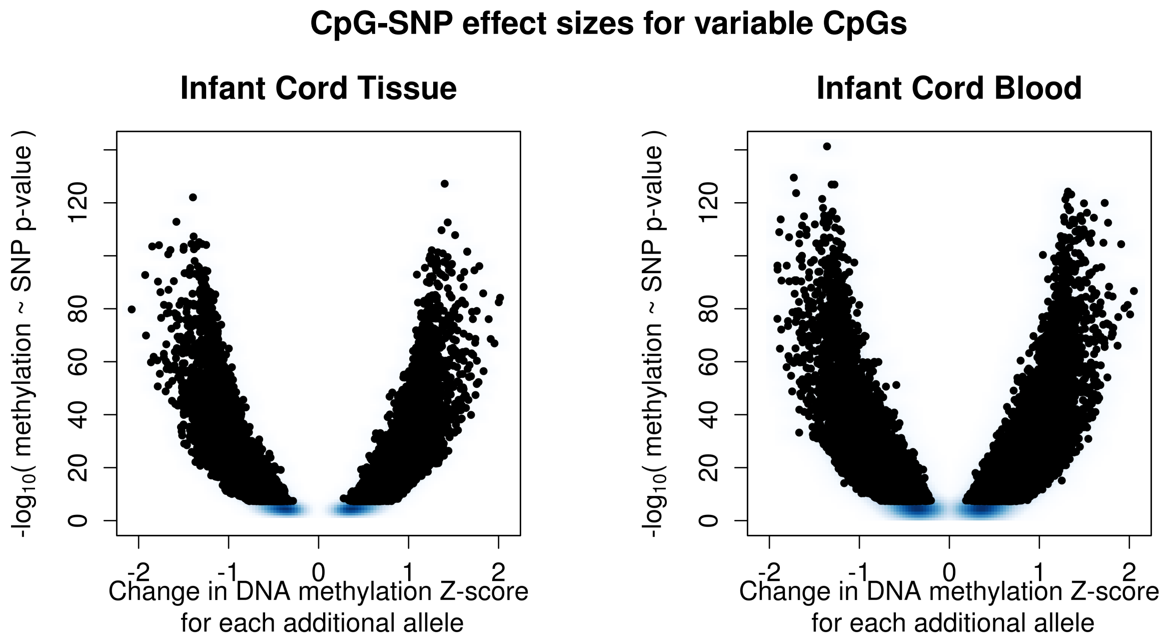


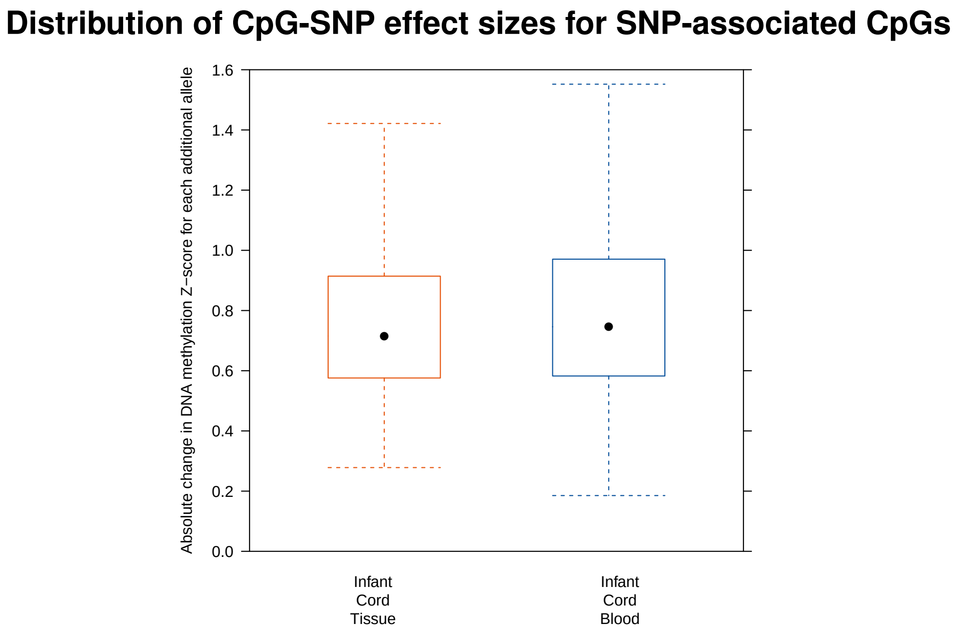


Hyperlink

Supplementary Table C2: **Replication of SNP-associated CpGs detected in each GUSTO infant tissue using the ALSPAC cohort.** Table shows the number of SNP-associated CpGs detected in each infant tissue in GUSTO, and whether the CpG was SNP-associated in infant cord blood in ALSPAC cohort (Gaunt *et al.*, 2016). A CpG was defined to be a SNP-associated in GUSTO if the most significant association between the CpG and *cis*-SNPs (all SNPs on the same chromosome as CpG) attained a p-value $< 5 \times 10^{-8}$, the commonly used bonferroni threshold for genome-wide association studies (corresponding to testing for $\sim 10^6$ independent SNPs across the genome at a family-wise Type 1 error rate of 0.05). A SNP-associated CpG was considered to be replicated in the ALSPAC cohort if the CpG had a CpG-SNP association p-value $< 5 \times 10^{-8}$ in the ALSPAC cohort and the reported CpG and SNP in the ALSPAC cohort are on the same chromosome. We did not require the SNP to be identical as the ALSPAC cohort used in this analysis comprised of Caucasian participants, while the GUSTO cohort comprised of Asian participants.

SNP-associated CpGs in GUSTO Infant Cord Tissue

| | Infant Cord Blood in ALSPAC |
|---|---|
| SNP-associated | 5965 (31%) |
| Not SNP-associated | 10130 (53%) |
| Not tested in ALSPAC | 3031 (16%) |
| Total | 19126 (100%) |

SNP-associated CpGs in GUSTO Infant Cord Blood

| | Infant Cord Blood in ALSPAC |
|---|---|
| SNP-associated | 9296 (54%) |
| Not SNP-associated | 5335 (31%) |
| Not tested in ALSPAC | 2505 (15%) |
| Total | 17136 (100%) |

Supplementary Figure C2: **SNPs explained a greater proportion of inter-individual variation in DNA methylation in infant cord blood (CB) than in infant cord tissue (CT): SNP-associated CpGs detected in each infant tissue, in a sensitivity analysis where we adjusted for surrogate variables.** Figure 3 shows the analysis adjusted for infant sex, gestational age, ethnicity, hospital, estimated cell-type proportions and other technical variables. This figure is analogous to Figure 3, except the analysis adjusted for infant sex, gestational age, ethnicity and surrogate variables; a surrogate variable analysis was conducted for each tissue separately, using all variable CpGs in each tissue, and the resulting surrogate variables can potentially capture cell-type composition and technical variables. **A -** Pie charts show the percentage of CpGs in each infant tissue whose inter-individual variation could be explained by SNPs (out of all CpGs which showed inter-individual variation in the infant tissue). A CpG whose inter-individual variation could be explained by SNPs (SNP-associated) was defined to be one where the most significant association between the CpG and *cis*-SNPs (all SNPs on the same chromosome as CpG) attained a p-value $< 5 \times 10^{-8}$, the commonly used Bonferroni threshold for genome-wide association studies (corresponding to testing for $\sim 10^6$ independent SNPs across the genome at a family-wise Type 1 error rate of 0.05). **B -** Overlap between SNP-associated, non-SNP-associated (but variable) and non-variable CpGs in the two tissues. Only CpGs which showed inter-individual variation in at least one tissue were included (N = 98,124). Examining each tissue separately, each of these 98,124 CpGs can either be SNP-associated, not SNP-associated or not variable in each tissue. The number of CpGs in each of these three sets in each tissue (SNP-associated, not SNP-associated or not variable) is shown in the bottom left bar chart (for each tissue the number of CpGs from the three sets will sum to 98,124). Collectively, the 98,124 CpGs can be grouped into 8 categories. The bottom right panel identifies each of these 8 categories, with the solid black dots representing the sets being considered. The top bar chart shows the number of CpGs in each of these 8 categories.

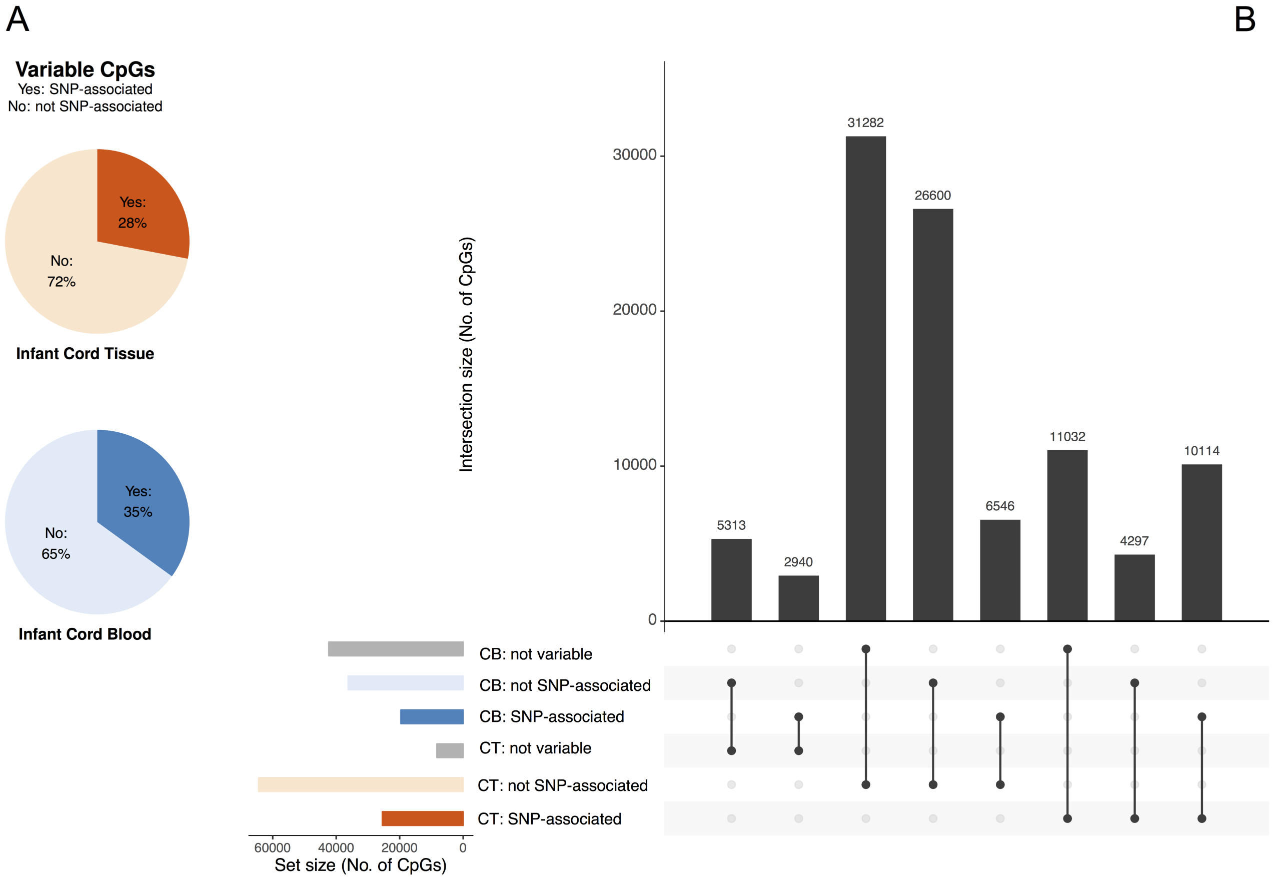

Supplementary Table D1: **CpGs whose inter-individual variation could be explained by prenatal factors in each infant tissue.** Table shows the number of CpGs in each tissue whose inter-individual variation could be explained by prenatal factors, and whether the CpG could be explained by prenatal factors in the other infant tissue. A CpG whose inter-individual variation could be explained by prenatal factors was defined to be one where the most significant association between the CpG and all 45 prenatal factors attained a p-value $< 1 \times 10^{-3}$, the bonferroni threshold for testing $45$ prenatal factors at a family-wise Type 1 error rate of 0.05.

| CpGs associated with prenatal factors in in infant cord tissue | |
|---|---|
| Associated with prenatal factors in infant cord blood | 95 (3%) |
| Not associated with prenatal factors in infant cord blood | 1567 (48%) |
| Not variable in infant cord blood | 1569 (49%) |
| Total | 3231 (100%) |

| CpGs associated with prenatal factors in infant cord blood | |
|---|---|
| Associated with prenatal factors in infant cord tissue | 95 (5%) |
| Not associated with prenatal factors in infant cord tissue | 1589 (79%) |
| Not variable in infant cord tissue | 318 (16%) |
| Total | 2002 (100%) |

Supplementary Table D2: **Overlap in prenatal-factor-associated and SNP-associated CpGs in each infant tissue.** Table shows the number of CpGs whose inter-individual variation could be explained by prenatal factors, and whether the CpG was also SNP-associated, in each infant tissue. A CpG whose inter-individual variation could be explained by prenatal factors was defined to be one where the most significant association between the CpG and all 45 prenatal factors attained a p-value $< 1 \times 10^{-3}$, the bonferroni threshold for testing $45$ prenatal factors at a family-wise Type 1 error rate of 0.05. A CpG was defined to be SNP-associated if the most significant association between the CpG and *cis*-SNPs (all SNPs on the same chromosome as CpG) attained a p-value $< 5 \times 10^{-8}$, the commonly used bonferroni threshold for genome-wide association studies (corresponding to testing for $\sim 10^6$ independent SNPs across the genome at a family-wise Type 1 error rate of 0.05).

CpGs associated with prenatal factors in infant cord tissue

| | |
|---|---|
| Associated with SNP | 708 (22%) |
| Not associated with SNP | 2523 (78%) |
| Total | 3231 (100%) |

CpGs associated with prenatal factors in infant cord blood

| | |
|---|---|
| Associated with SNP | 633 (32%) |
| Not associated with SNP | 1369 (68%) |
| Total | 2002 (100%) |

Supplementary Figure D1: **Effect sizes for CpG-prenatal-factor associations in the two infant tissues.** Boxplot shows the distribution of (absolute values of) effect-sizes of CpG-prenatal-factor associations for all CpG-prenatal-factor associations with p-values $< 1 \times 10^{-3}$. Effect sizes are represented as change in DNA methylation Z-score for two standard deviations increase in prenatal factor (for continuous prenatal factor variables) or for comparing two categories of prenatal factor (for binary prenatal factor variables).

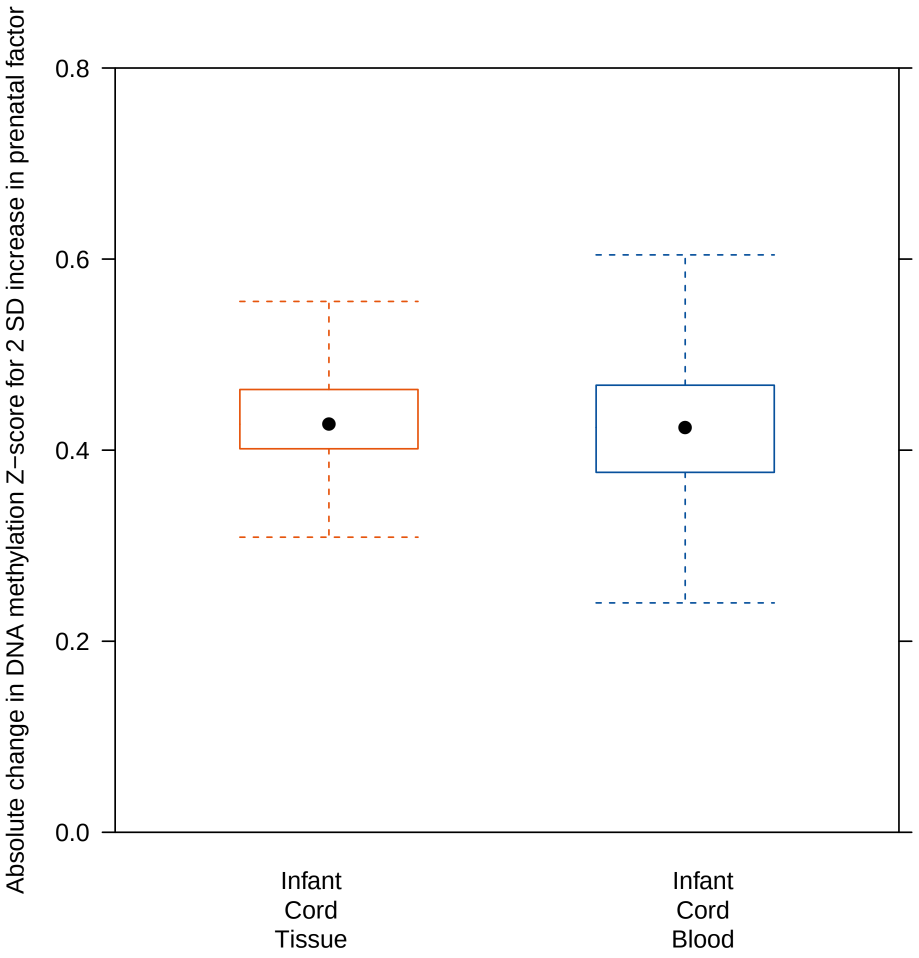

Supplementary Figure D2: **(Lack of) concordance in EWAS results from the two infant tissues: number of CpGs associated with individual prenatal factors in infant cord tissue.** Bar chart shows the number of CpGs associated with each prenatal factor (CpG-prenatal-factor p-value was $< 1 \times 10^{-3}$) in infant cord tissue, and whether the CpG was associated with the same prenatal factor in infant cord blood.

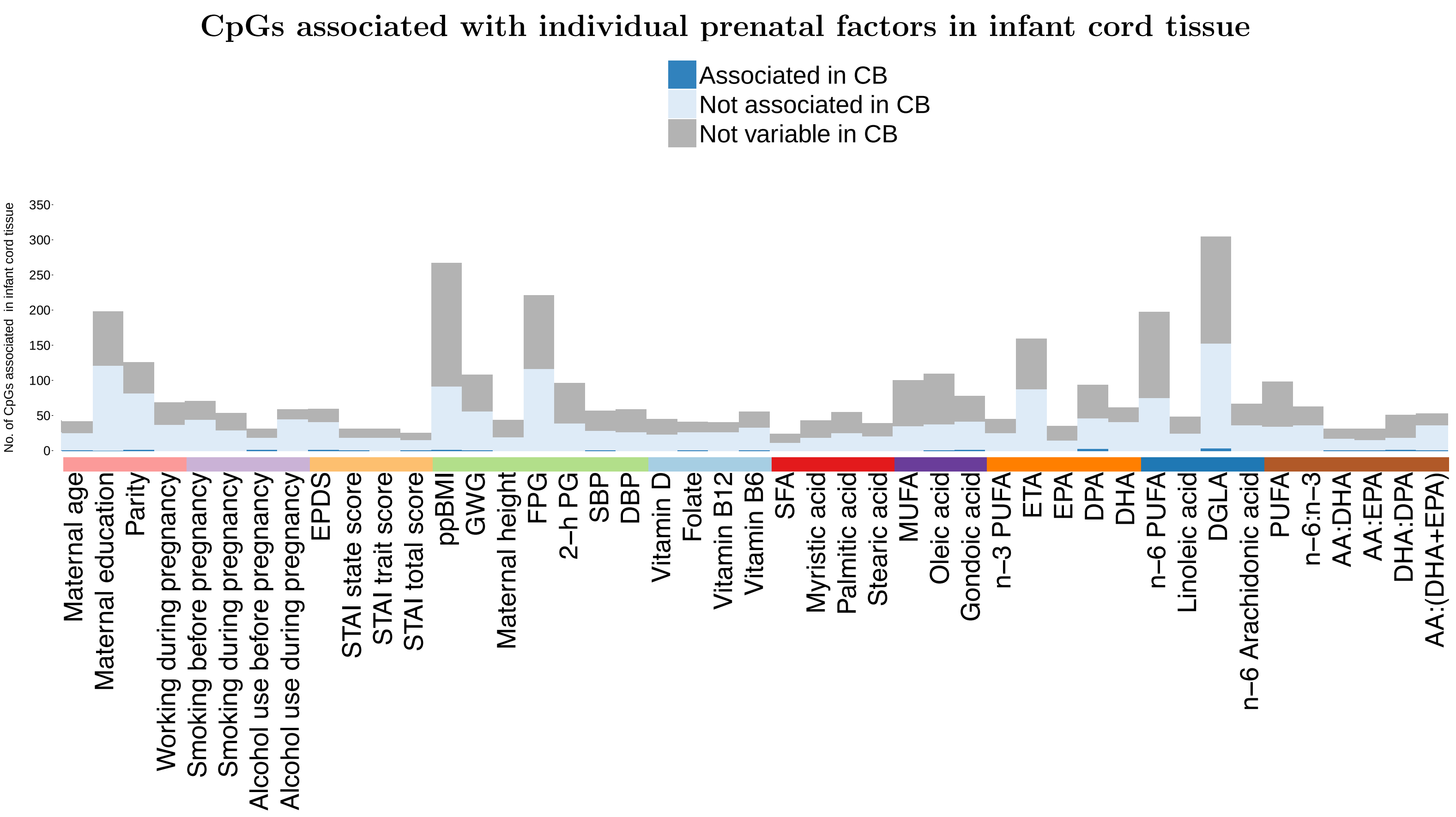

Supplementary Figure D3: **(Lack of) concordance in EWAS results from the two infant tissues: number of CpGs associated with individual prenatal factors in infant cord blood.** Bar chart shows the number of CpGs associated with each prenatal factor (CpG-prenatal-factor p-value was $< 1 \times 10^{-3}$) in infant cord blood, and whether the CpG was associated with the same prenatal factor in infant cord tissue.

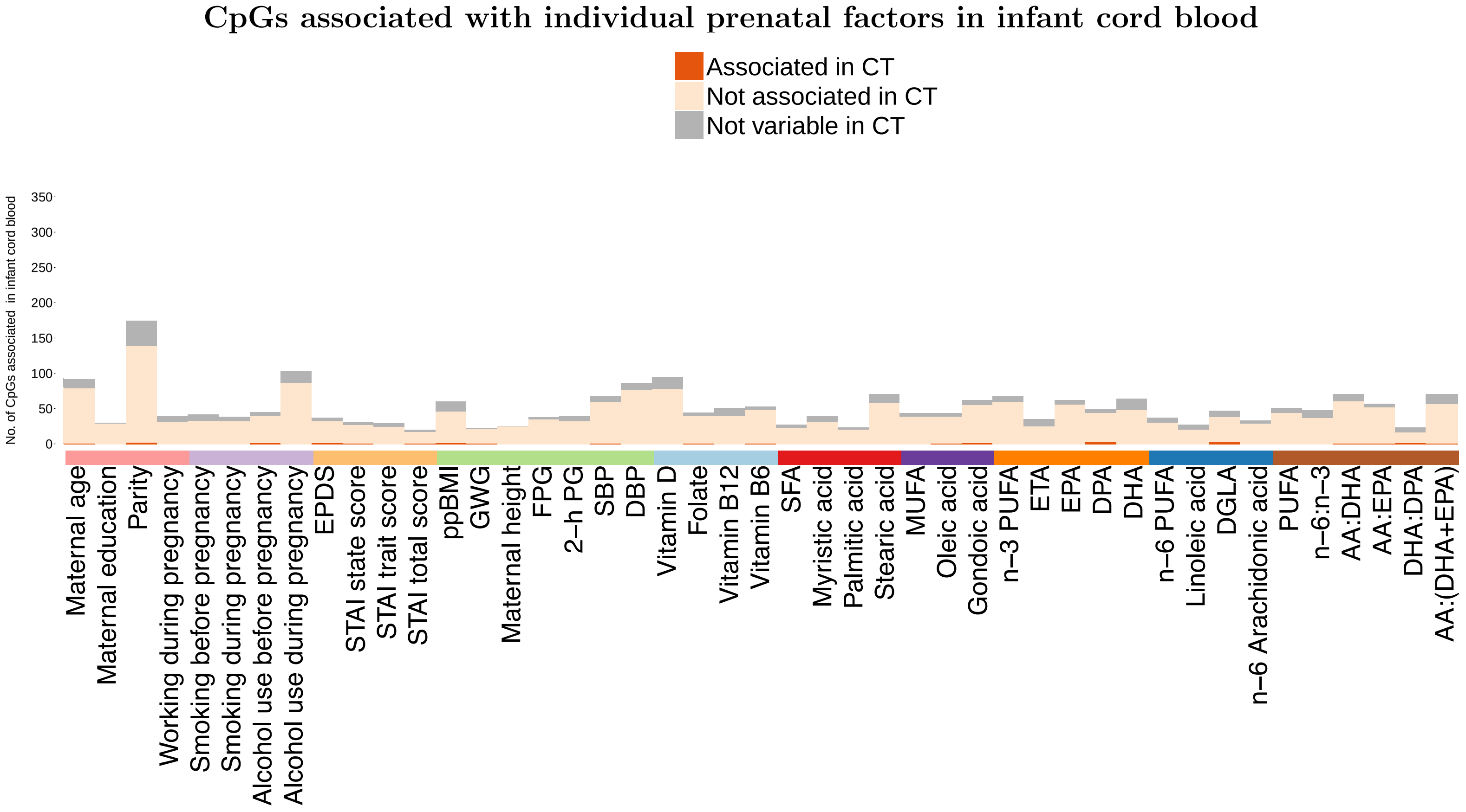


Hyperlink

Supplementary Figure D4: **Prenatal factors (PFs) explained a similar proportion of inter-individual variation in infant cord blood (CB) and infant cord tissue (CT): CpGs where the inter-individual variation in DNA methylation were explained by PFs, in a sensitivity analysis adjusted for surrogate variables.** Figure 4 shows the analysis adjusted for infant sex, gestational age, ethnicity, hospital, estimated cell-type proportions and other technical variables. This figure is analogous to Figure 4, except the analysis adjusted for infant sex, gestational age, ethnicity and surrogate variables; a surrogate variable analysis was conducted for each tissue separately, using all variable CpGs in each tissue, and the resulting surrogate variables can potentially capture cell-type composition and technical variables. Pie charts (left panel) show the percentage of CpGs in each infant tissue whose inter-individual variation could be explained by PFs (out of all CpGs which showed inter-individual variation in the infant tissue). A CpG whose inter-individual variation could be explained by PFs was defined to be one where the most significant association between the CpG and all 45 PFs attained a p-value $< 1 \times 10^{-3}$, the Bonferroni threshold for testing $45$ PFs at a family-wise Type 1 error rate of 0.05. Right panel shows overlap between PF-associated, non-PF-associated (but variable) and non-variable CpGs in the two tissues. Only CpGs which showed inter-individual variation in at least one tissue were included (N = 98,124). Examining each tissue separately, each of these 98,124 CpGs can either be PF-associated, non-PF-associated or not variable in each tissue. The number of CpGs in each of these three sets in each tissue is shown in the bottom left bar chart. Collectively, the 98,124 CpGs can be grouped into 8 categories. The bottom right panel identifies each of these 8 categories, with the solid black dots representing the sets being considered. The top bar chart shows the number of CpGs in each of these 8 categories.

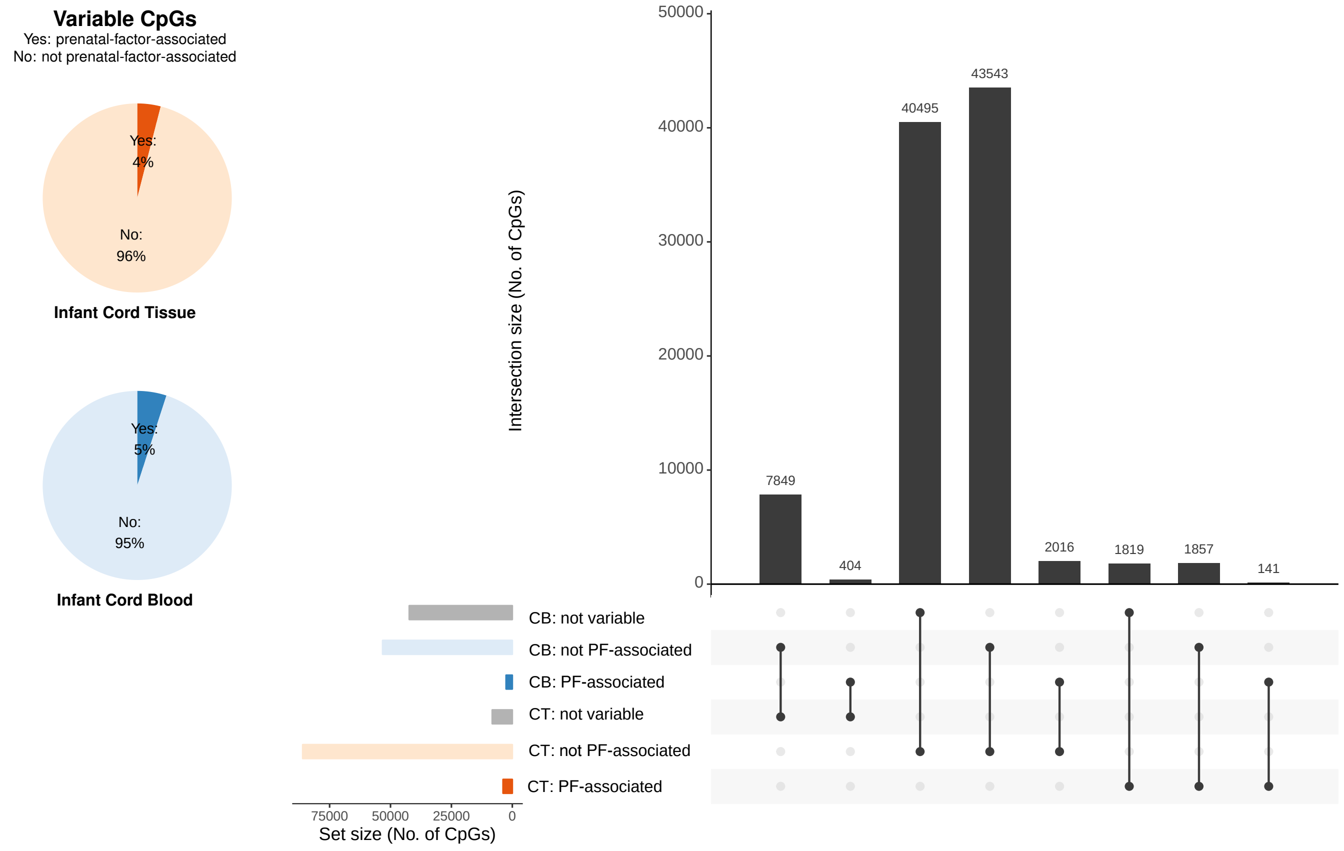

Supplementary Figure D5: **(Lack of) concordance in EWAS results from the two infant tissues: number of CpGs associated with individual prenatal factors in infant cord tissue, in a sensitivity analysis adjusted for surrogate variables.** Supplementary Figure D2 shows the analysis adjusted for infant sex, gestational age, ethnicity, hospital, estimated cell-type proportions and other technical variables. This figure is analogous to Supplementary Figure D2, except the analysis adjusted for infant sex, gestational age, ethnicity and surrogate variables; a surrogate variable analysis was conducted for each tissue separately, using all variable CpGs in each tissue, and the resulting surrogate variables can potentially capture cell-type composition and technical variables. Bar chart shows the number of CpGs associated with each prenatal factor (CpG-prenatal-factor p-value was $< 1 \times 10^{-3}$) in infant cord tissue, and whether the CpG was associated with the same prenatal factor in infant cord blood.

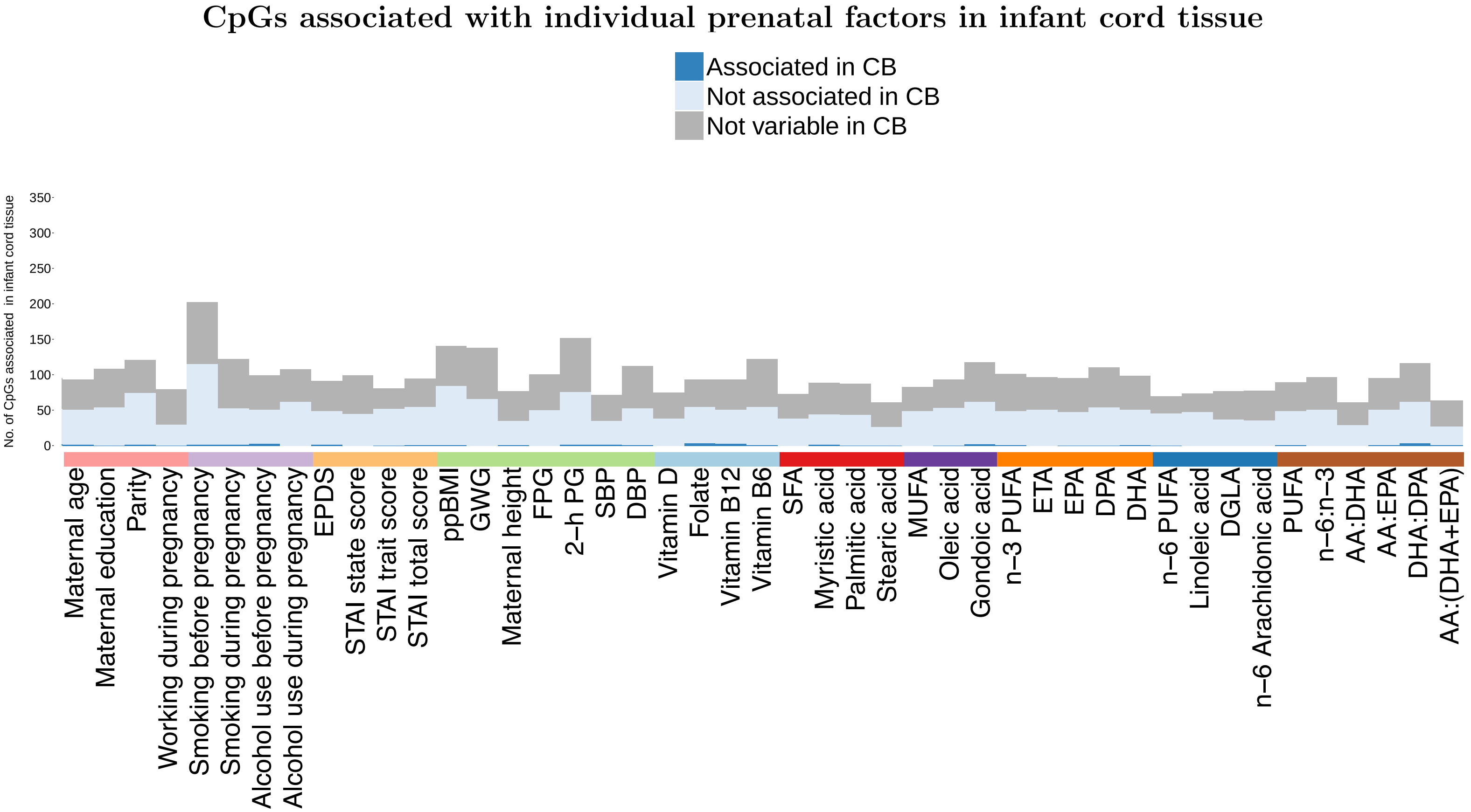


Hyperlink

Supplementary Figure D6: **(Lack of) concordance in EWAS results from the two infant tissues: number of CpGs associated with individual prenatal factors in infant cord blood, in a sensitivity analysis adjusted for surrogate variables.** Supplementary Figure D3 shows the analysis adjusted for infant sex, gestational age, ethnicity, hospital, estimated cell-type proportions and other technical variables. This figure is analogous to Supplementary Figure D3, except the analysis adjusted for infant sex, gestational age, ethnicity and surrogate variables; a surrogate variable analysis was conducted for each tissue separately, using all variable CpGs in each tissue, and the resulting surrogate variables can potentially capture cell-type composition and technical variables. Bar chart shows the number of CpGs associated with each prenatal factor (CpG-prenatal-factor p-value was $< 1 \times 10^{-3}$) in infant cord blood, and whether the CpG was associated with the same prenatal factor in infant cord tissue.

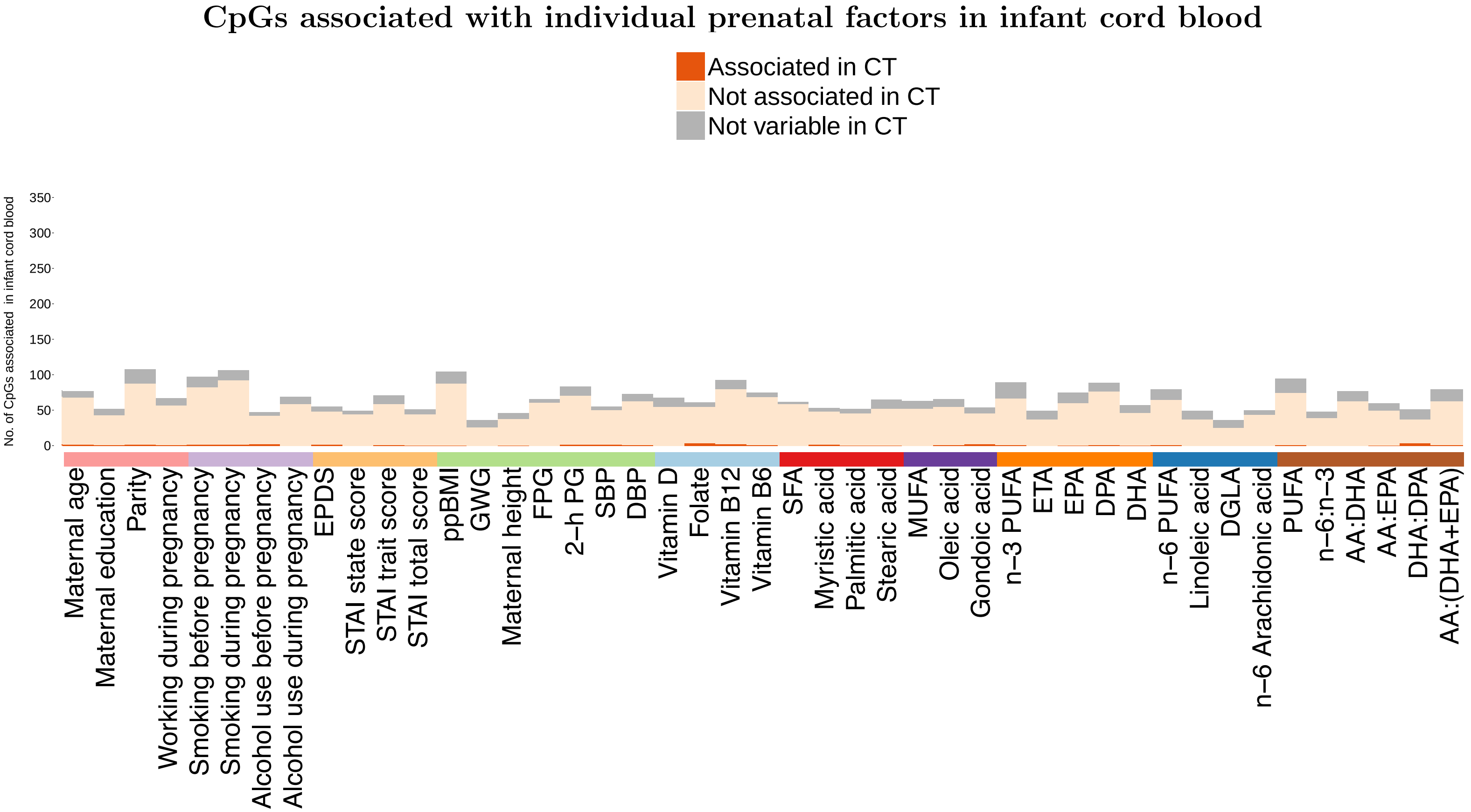

Supplement: Supplementary file 1 — Supplementary tables and figures. (PDF 2720 kb) [file 12916_2017_970_MOESM1_ESM.pdf]
