# Supplementary material for: Choice of surrogate tissue influences neonatal EWAS findings
Source: BMC Med. 2017 Dec 5;15:211. doi: 10.1186/s12916-017-0970-x (PMC5715509; doi:10.1186/s12916-017-0970-x)
Supplement: Supplementary file 4 — Information on using DNA methylation data in Additional files 2 and 3. (PDF 36 kb) [file 12916_2017_970_MOESM4_ESM.pdf]

Choice of surrogate tissue influence neontatel EWAS findings

Information on DNA methylation file

- Filename for cord tissue:
  v102bdata_GUSTO_InfantCord_450k_295samples-commonCpGs.txt
  (md5sum: 659fe232ea5de463cf41460bf070246b)
- Flename for cord blood:
  v102bdata_GUSTO_InfantBlood_450k_295samples-commonCpGs.txt
  (md5sum: f5a89debc501811e074498b5196d7894)
- Each tab-delimited file contains methylation values for 295 samples, 239560 CpGs.
- Each row is a CpG, each column is a sample, first column gives CpG name.
